# Agronomic, Hormonal, and Seed Quality Responses of Soybeans to Four Spray Treatment Regimes Under Irrigated Conditions in Xinjiang: A Three-Year Field Study

**DOI:** 10.3390/plants15162528

**Published:** 2026-08-20

**Authors:** Hao Cheng, Yiqun Wang, Hao Wang, Gulisumuayi Maimaiti, Qi Han, Xinna Zheng, Xinghu Song, Qiang Zhao

**Affiliations:** College of Agriculture, Xinjiang Agricultural University, Urumqi 830052, China; henrycheng24@163.com (H.C.); wangzimoc429@163.com (Y.W.); wh79292@163.com (H.W.); 19018264647@163.com (G.M.); seveny0801@163.com (Q.H.); 15739276613@163.com (X.Z.)

**Keywords:** soybean, plant growth regulators, yield formation, total above-ground biomass partitioning, phytohormones, source–sink relationships

## Abstract

Soybean production commonly faces challenges including severe pod abscission, incomplete seed filling, and uncoordinated source–sink relationships that limit yield potential. Plant growth regulator application may influence source–sink relationships and thereby affect soybean (*Glycine max* (L.) Merr.) yield formation. This three-year field study (2023–2025) evaluated the effects of four spray programs on soybean yield, quality, total above-ground biomass partitioning, and endogenous hormone dynamics under Xinjiang’s irrigated production conditions. The four treatments were an untreated control (CK), naphthaleneacetic acid alone (NAA; 300 g ha^−1^), naphthaleneacetic acid plus prohexadione-calcium (NPC; 300 + 450 g ha^−1^), and naphthaleneacetic acid plus prohexadione-calcium and iron chlorin e6 (NCE; 300 + 450 + 45 g ha^−1^). The spray programs were applied at the fourth-trifoliolate and full-pod stages. Results showed that NCE treatment consistently produced the greatest yield increases (13.1–14.4%) compared with the control. This response was associated with greater middle-node pod retention, increased 100-seed weight (3.6–6.4%), and improved reproductive organ biomass allocation (44.7–53.3% at maturity vs. 40.9–45.7% in controls). NCE significantly elevated leaf trans-zeatin content (37.1–91.9%) within 24 h after application and improved seed protein concentration by up to 11.2%, while seed residues of all applied compounds remained well below safety thresholds (<0.05 mg kg^−1^). Correlation analysis revealed strong positive relationships between trans-zeatin levels and both reproductive biomass allocation (*r* = 0.74–0.90, *p* < 0.01) and grain yield (*r* = 0.86–0.96, *p* < 0.01). These findings indicate that NCE was the best-performing of the four tested spray programs under Xinjiang’s irrigated production conditions, although the individual contributions and possible interactions of the three compounds require further factorial evaluation.

## 1. Introduction

Soybeans (*Glycine max* (L.) Merr.) hold a strategic position within the global agricultural landscape, functioning as a major source of plant protein and vegetable oil for food supply and livestock feed. Owing to its remarkable nutritional attributes, it remains essential to both primary agricultural systems and the wider agro-industrial economy [1]. Xinjiang, recognized as one of the high-yield regions for soybean cultivation in China, recorded in 2025 a yield advantage of 542.21 kg ha^−1^ above the national average [2]. Soybean cultivation is influenced by both inherent varietal characteristics and various abiotic stress factors, which can result in adverse outcomes such as flower and pod abscission, incomplete seed filling, and inefficient carbohydrate translocation [3,4,5]. In Xinjiang, these constraints collectively prevent the crop from reaching its optimal yield potential, even under favorable agronomic conditions in certain areas. Conventional cultivation practices, such as regulating planting density and irrigation volume, are often ineffective in promptly mitigating sudden abiotic stresses, including short-term heatwaves and soil salinity [6,7]. Such limitations highlight the urgent need to identify and develop efficient and easily deployable strategies that can safeguard crop performance under rapidly changing environmental conditions.

The judicious application of plant growth regulators (PGRs) has been widely recognized as a convenient and effective agronomic approach to enhance crop yield and improve product quality by modulating physiological and biochemical processes, such as cell division, elongation, and nutrient translocation [8,9,10]. Moreover, PGRs can contribute to greater stress resilience, enabling crops to maintain productivity under suboptimal environmental conditions [11,12]. Naphthaleneacetic acid (NAA), a synthetic auxin, has been applied in soybean production to reduce flower and pod abscission during critical reproductive stages. Recent field studies demonstrated that NAA application increased seed number per plant by 9.3% to 13.1% compared with untreated controls [13,14]. However, auxin application alone may stimulate excessive vegetative growth, which can divert carbohydrate resources from reproductive organs and limit potential yield improvements [15].

Prohexadione-calcium (Pro-Ca) has been associated with the inhibition of late steps in gibberellin biosynthesis, although the specific metabolic steps affected may vary among plant species and experimental conditions. This compound was initially developed for fruit tree management to control vegetative shoot growth and improve fruit quality [16,17]. Recent research extended Pro-Ca application to field crops. Feng et al. reported that Pro-Ca application in soybeans grown under saline-alkali stress increased seed yield through improved ion homeostasis, enhanced antioxidant defense, and maintained photosynthetic capacity [18]. Previous studies have proposed that auxin- and anti-gibberellin-based spray programs may influence the allocation of resources between vegetative and reproductive organs [19], although this possibility requires evaluation using appropriate combination treatments.

Iron chlorin e6 (ICE-6) represents a semi-synthetic chlorophyll derivative containing iron instead of magnesium in the porphyrin ring structure. This modification alters the spectral absorption properties of the molecule, enabling broader light absorption capacity across the visible spectrum compared to natural chlorophyll [20]. The compound also exhibits greater stability under high irradiance and elevated temperature conditions [21]. Greenhouse studies in vegetable crops indicated that foliar ICE-6 application enhanced net photosynthetic rate and improved carbon assimilation efficiency [22]. The inclusion of ICE-6 in growth-regulator spray programs may influence carbon assimilation and yield formation [23], but its contribution within multicomponent programs requires experimental evaluation.

Source–sink relationships determine seed yield formation in soybean. Source capacity depends on leaf area, photosynthetic rate, and total biomass accumulation, whereas sink strength depends on the number of pods and seeds, as well as individual seed growth rate [24]. Phytohormones mediate communication between source and sink organs, regulating processes such as cell division, nutrient allocation, and senescence [25]. Cytokinins promote cell division in developing seeds and delay leaf senescence [26]. Auxins regulate pod initiation and seed development [27,28]. Gibberellins control stem elongation and influence flower formation [29]. Abscisic acid modulates stress responses and participates in seed maturation [30]. Evaluating changes in endogenous hormone levels following the application of plant growth regulators may provide insight into physiological processes associated with yield formation [9,31]. Measuring short-term hormone responses in leaves can further characterize the hormonal responses associated with the tested spray programs [26,31].

Previous studies have separately examined the agronomic or physiological effects of NAA [13,14,32,33], Pro-Ca [18,34,35], and ICE-6 [22,36,37]. However, soybean responses to sequential NAA-based spray programs containing Pro-Ca, with or without ICE-6, remain insufficiently characterized under the agro-climatic conditions of Xinjiang. In particular, limited information is available regarding differences among these spray programs in yield formation, biomass partitioning, nutrient accumulation, seed quality, and short-term endogenous hormone responses. Multi-year field evidence under continental irrigated production conditions is also limited, and the consistency of treatment responses across growing seasons requires evaluation. Moreover, it remains unclear whether these spray programs result in residues of the applied compounds in harvested soybean seeds at levels relevant to food safety. Because the treatment set used in this study was not a complete factorial design, the study was intended to compare the four tested spray programs rather than to estimate the independent main effects or interactions of the three compounds.

Based on these considerations, we hypothesized that soybean yield, seed quality, total above-ground biomass allocation, and endogenous hormone profiles would differ among the four tested spray programs and that the NCE program would produce the greatest agronomic response under Xinjiang’s irrigated production conditions. Accordingly, this study compared four specific spray programs and examined the agronomic, physiological, and hormonal responses associated with their application. The specific objectives were to (i) compare soybean yield and seed quality among the untreated control, NAA alone, NAA + Pro-Ca, and NAA + Pro-Ca + ICE-6 spray programs across three growing seasons; (ii) compare pod distribution along the main stem, total above-ground biomass partitioning between vegetative and reproductive organs, and nutrient accumulation dynamics among these programs during reproductive development; and (iii) characterize short-term endogenous hormone responses in leaves following application of the tested programs and examine their associations with yield and total above-ground biomass allocation. Additionally, residue levels of the applied compounds in harvested seeds were determined and compared with the relevant safety thresholds. This study provides multi-year field evidence of the agronomic, physiological, and hormonal responses associated with the four tested spray programs in soybean production systems in Northwest China.

## 2. Results

### 2.1. Effects of Treatments on Yield and Yield Components

Treatment did not significantly affect plant population, which remained within 41.2–42.8 × 10^4^ plants ha^−1^ across the three years (Table 1). Year, treatment, and their interaction were also nonsignificant, indicating that treatment effects on yield and yield components were not attributable to differences in plant population.

Total pod number per plant, including pods borne on both the main stem and branches, was significantly affected by treatment (*p* < 0.01; Table 1). Relative to CK, NAA, NPC, and NCE increased total pod number per plant by 7.8–32.0%. In each year, all three foliar treatments produced significantly more total pods per plant than CK, whereas no significant differences were detected among NAA, NPC, and NCE. Seed number per plant was significantly affected by year and treatment (*p* < 0.01). Compared with CK, the treatments increased seed number by 8.1–15.0%, with NPC showing the largest numerical increase in 2023 and 2025. The year × treatment interaction was nonsignificant, indicating no detectable variation in treatment responses among years at the study site. Given the single location and only three annual environments, this result should not be interpreted as evidence of seed-number stability across environments. Treatment significantly affected 100-seed weight (*p* < 0.01). NCE consistently produced the highest numerical values, increasing 100-seed weight by 3.6–6.4% relative to CK. NAA had little effect, whereas NPC significantly increased 100-seed weight only in 2024. Yield was significantly affected by year and treatment (*p* < 0.01). Mean yields of CK, NAA, NPC, and NCE were 4546.20, 4910.12, 5060.99, and 5143.33 kg ha^−1^ in 2023; 4997.55, 5448.64, 5579.67, and 5714.73 kg ha^−1^ in 2024; and 4792.14, 5300.19, 5393.64, and 5479.61 kg ha^−1^ in 2025, respectively.

All treatments increased yield relative to CK. Yield increases were 8.00–10.60% for NAA, 11.32–12.55% for NPC, and 13.13–14.35% for NCE. Although NCE had the highest numerical yield in each year, planned contrasts did not support its consistent statistical superiority over NPC. The NCE–NPC difference was significant only in 2024, when NCE increased yield by 135.06 kg ha^−1^ (95% CI: 11.56–258.56 kg ha^−1^); the contrasts were nonsignificant in 2023 and 2025. Similarly, NPC was numerically higher-yielding than NAA in all years, but none of the NPC–NAA contrasts was significant. In 2025, NPC did not differ significantly from either NCE or NAA.

By contrast, the NAA–CK, NPC–CK, and NCE–CK contrasts were positive in all years, with 95% CIs excluding zero, confirming the yield advantage of each treatment over CK (Table 2). The year × treatment interaction for yield was nonsignificant, indicating no detectable differences in treatment responses among the three years at the study site. However, this result does not establish yield stability or broad environmental adaptability because the experiment involved only one location and three annual environments.

### 2.2. Effects of Treatments on Seed Residues and Quality

**Figure 1:**
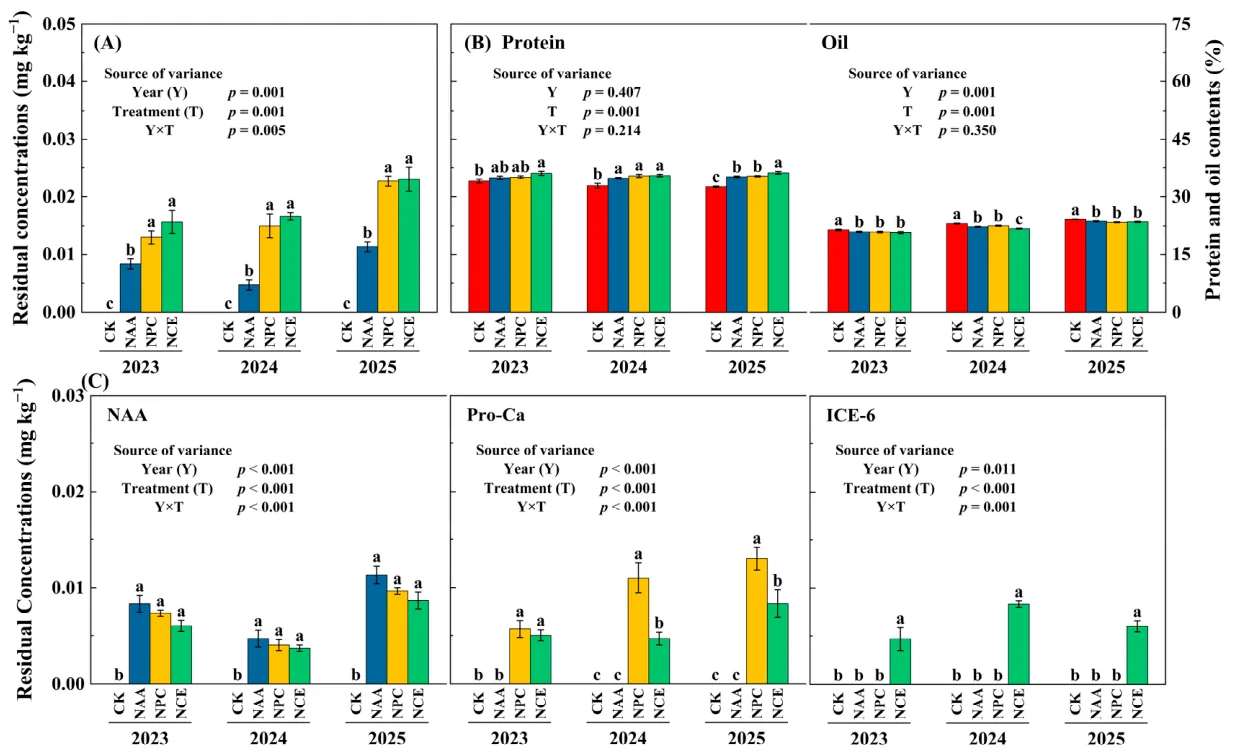
Effects of plant growth regulator treatments on (**A**) residual concentrations in mixed-spray programs, (**B**) seed quality, and (**C**) residues of individual plant growth regulators (NAA, Pro-Ca, and ICE-6) across three growing seasons (2023–2025). ND (not detected): NAA in CK; Pro-Ca in CK and NAA; ICE-6 in CK, NAA, and NPC. All quantified values were above the respective LOQ. Error bars represent the standard error of the mean. Different lowercase letters in the vertical direction indicate significant differences between treatments in the same year (*p* < 0.05).

Seed residue, seed protein concentration, and seed oil concentration showed different responses to the treatments (Figure 1). Individual analyte residues varied by spray program and year (Figure 1C). NAA residues were detected in all NAA-containing treatments (NAA, NPC, and NCE) but not in the control, ranging from 0.004 to 0.011 mg kg^−1^ across the three years. Pro-Ca residues were detected only in treatments where Pro-Ca was applied (NPC and NCE), ranging from 0.005 to 0.013 mg kg^−1^, with the highest concentrations observed in NPC treatments in 2024 and 2025 (0.011 and 0.013 mg kg^−1^, respectively). ICE-6 residues were detected exclusively in NCE treatments, ranging from 0.005 to 0.008 mg kg^−1^, with the peak value in 2024. All three analytes were non-detectable in the untreated control across all three years.

Year, treatment, and their interaction significantly affected residue levels for all three plant growth regulators. For NAA, the effects of year, treatment, and their interaction were all highly significant (*p* < 0.001). Similarly, Pro-Ca residues showed highly significant effects of year (*p* < 0.001), treatment (*p* < 0.001), and their interaction (*p* < 0.001). ICE-6 residues were significantly influenced by year (*p* = 0.011), treatment (*p* < 0.001), and their interaction (*p* = 0.001). All measured residue concentrations remained below 0.05 mg kg^−1^, well within regulatory limits.

Seed protein concentration was mainly affected by treatment. NCE showed the strongest effect on protein improvement, increasing seed protein concentration by 5.6%, 7.9%, and 11.2% compared with CK from 2023 to 2025, respectively (Figure 1B). Seed oil concentration showed a consistent decrease after treatment application. CK had the highest oil concentration in each year, while the treated plants showed a 2.1% to 5.6% decrease compared with CK. Year and treatment significantly affected seed oil concentration, but their interaction was not significant.

### 2.3. Effects of Treatments on Pod Number and Pod Spatial Distribution

**Figure 2:**
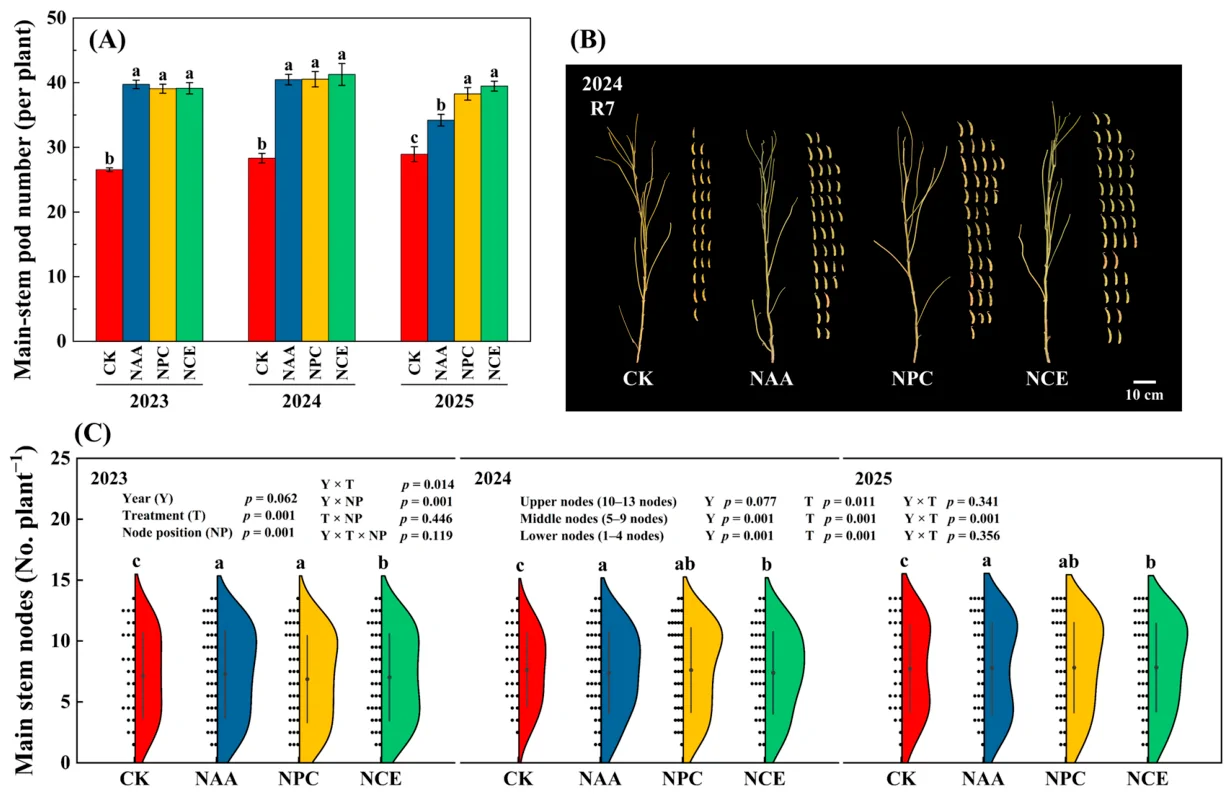
Effects of plant growth regulator treatments on main-stem effective pod number (**A**), pod phenotype (**B**), and pod distribution across plant nodes (**C**) at the R7 stage (2023–2025). The data points in (**C**) represent the number of pods corresponding to each node position. Different lowercase letters in the vertical direction indicate significant differences between treatments in the same year (*p* < 0.05).

Pod number at the R7 stage increased following treatment application (Figure 2A). CK had the lowest pod number in each year, ranging from 26.53 to 28.93 pods plant^−1^. In 2023, NAA, NPC, and NCE increased pod number by 49.8%, 47.3%, and 47.5%, respectively, compared with CK, with no significant differences among the three treatments. In 2024 and 2025, pod numbers under NPC and NCE were higher than those under NAA. Compared with CK, NCE increased pod number by 45.7% and 36.4% in 2024 and 2025, respectively, whereas NPC increased it by 43.1% and 32.3%. NAA also increased pod number, although its effects were smaller than those of NPC and NCE in these two years. Overall, NPC and NCE consistently increased pod number across the three years, although the magnitude of the treatment response varied among years.

Mixed-effects analysis of node-level pod distribution revealed significant main effects of treatment (*p* < 0.001) and node position (*p* < 0.001), whereas the main effect of year was not significant (*p* = 0.052). Significant treatment × year (*p* = 0.014) and node position × year (*p* < 0.001) interactions were detected. In contrast, neither the treatment × node position interaction (*p* = 0.446) nor the treatment × node position × year interaction (*p* = 0.199) was significant. These results provide no statistical evidence that treatment effects differed among node positions or that such differences varied among years (Figure 2C).

Region-specific summaries indicated that pod number generally increased following treatment application in the lower (nodes 1–4), middle (nodes 5–9), and upper (nodes 10–13) canopy regions, although the magnitude of the observed increases varied descriptively among regions and years. In the lower region, both year and treatment had significant effects (both *p* < 0.001), whereas the year × treatment interaction was not significant (*p* = 0.356). In the middle region, the effects of year and treatment and their interaction were all significant (all *p* < 0.001). For example, in 2024, pod number in the middle region was 20.0 pods plant^−1^ under NCE, compared with 14.0 pods plant^−1^ under CK. In 2025, NCE and NPC increased pod number in the middle region by 60% and 40%, respectively, relative to CK. In the upper region, treatment had a significant effect (*p* = 0.011), whereas neither the effect of year (*p* = 0.077) nor the year × treatment interaction (*p* = 0.341) was significant. These region-specific analyses characterize treatment effects within individual canopy regions but do not provide statistical evidence that the treatment response in the middle region differed from that in the lower or upper regions.

### 2.4. Effects of Treatments on Total Above-Ground Biomass Accumulation and Allocation

**Figure 3:**
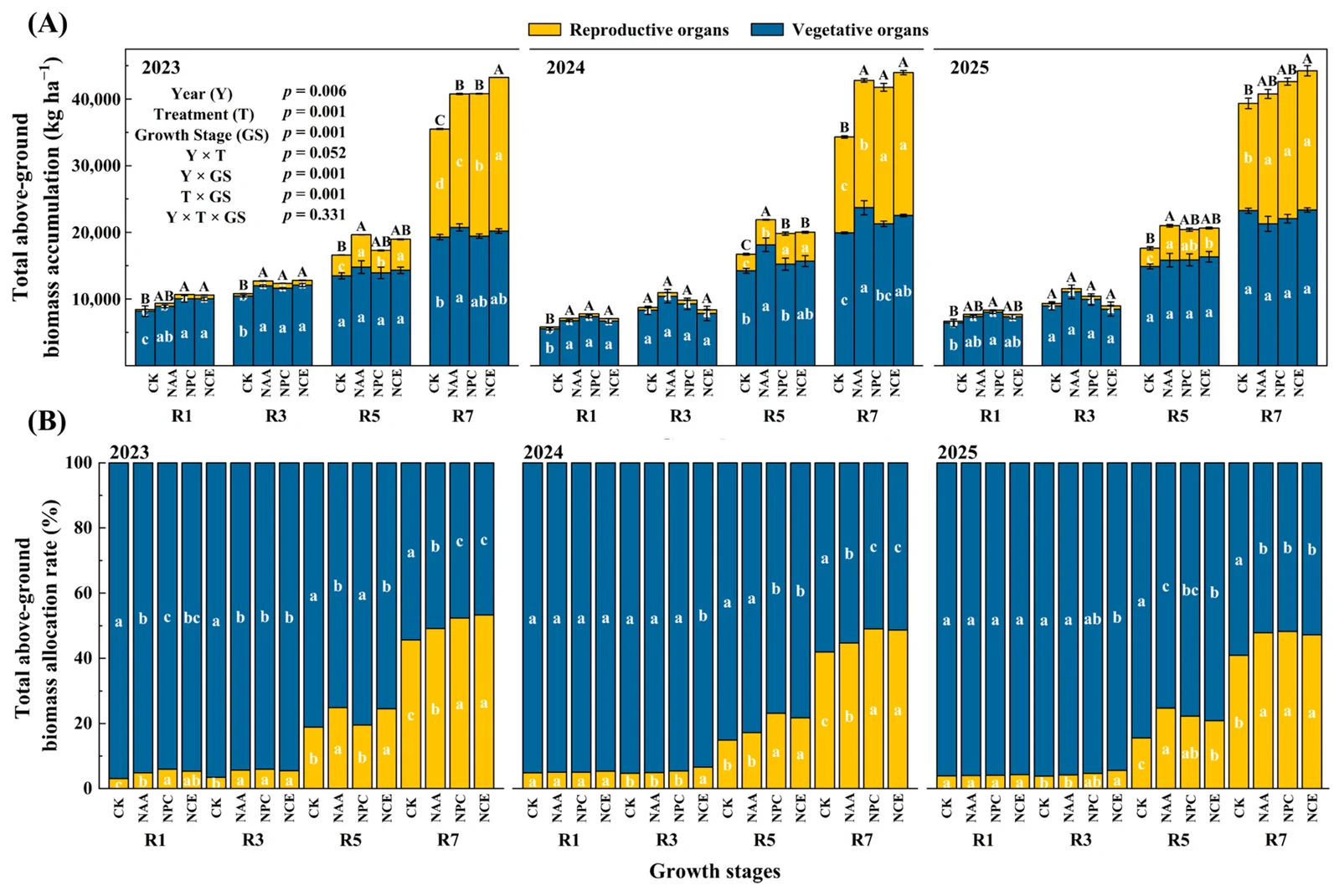
Effects of plant growth regulator treatments on total above-ground biomass accumulation (**A**) and allocation (**B**) in soybean vegetative and reproductive organs at the R1, R3, R5, and R7 growth stages from 2023 to 2025. Different lowercase and uppercase letters above bars indicate significant differences among treatments within the same growth stage in the same year at *p* < 0.05.

Treatments altered total above-ground biomass accumulation and its partitioning during reproductive growth (Figure 3). Mixed-effects analysis showed significant effects of year (*p* = 0.006), treatment (*p* = 0.001), and growth stage (*p* = 0.001) on total above-ground biomass. The year × treatment interaction was not significant (*p* = 0.052), whereas the year × growth stage and treatment × growth stage interactions were significant (both *p* = 0.001). The year × treatment × growth stage interaction was not significant (*p* = 0.331). These results indicate that total above-ground biomass varied among years, treatments, and growth stages, and that the magnitude of the treatment response depended on growth stage. Total above-ground biomass accumulation across growth stages also differed among years; however, the absence of a significant three-way interaction indicates that the treatment-dependent pattern across growth stages did not differ significantly among years.

At R1, total above-ground biomass under NAA, NPC, and NCE was 10.9–33.9% higher than that under CK across the three years. Reproductive organ dry mass showed larger proportional differences than vegetative organ dry mass at this stage. Compared with CK, reproductive organ dry mass was 30.5–146.6% higher under NPC and 25.4–118.9% higher under NCE. These descriptive comparisons suggest that the treatment-associated differences at R1 were particularly evident in the reproductive fraction, with NPC generally showing the largest increase. At this stage, reproductive organ dry mass comprised retained flower buds, open flowers, and any visible young reproductive structures present on the sampled plants at harvest; abscised reproductive material was excluded.

At R3, reproductive organ dry mass was higher under all three treatments than under CK in each of the three years. Relative to CK, reproductive organ dry mass was 28.0–94.4%, 29.0–98.8%, and 32.3–89.6% higher under NAA, NPC, and NCE, respectively. The corresponding differences in total above-ground biomass were generally smaller. NAA and NPC increased total above-ground biomass by 17.4–25.4% and 11.7–14.1%, respectively. NCE resulted in higher total above-ground biomass than CK in 2023 but slightly lower values in 2024 and 2025, coinciding with lower vegetative organ dry mass in those two years.

At R5, treatment-associated differences in biomass were more pronounced. Compared with CK, total above-ground biomass was 19.3–30.9%, 11.6–18.5%, and 17.1–22.4% higher under NAA, NPC, and NCE, respectively. Reproductive organ dry mass was 50.9–89.7% higher under NAA, 7.8–84.5% higher under NPC, and 49.0–75.0% higher under NCE. Among the treatments, NAA had the highest reproductive organ dry mass in 2023 and 2025, whereas NPC and NCE showed comparatively higher values in 2024.

At R7, differences in total above-ground biomass were mainly associated with differences in reproductive organ dry mass. NCE produced the highest total above-ground biomass among the treatments in all three years, with values 12.4–28.2% higher than those under CK. Total above-ground biomass under NAA and NPC was 3.6–24.8% and 8.3–21.8% higher than that under CK, respectively. NCE also produced the highest reproductive organ dry mass in all three years, exceeding CK by 29.7–49.1%.

Biomass partitioning at R7 showed that reproductive organs accounted for 40.9–45.7% of total above-ground biomass under CK, compared with 44.7–53.3% under the three treatments. NPC and NCE generally exhibited higher reproductive organ allocation than NAA. Overall, the descriptive stage-specific results suggest that the treatments were associated with greater reproductive organ dry mass and a higher proportion of total above-ground biomass allocated to reproductive organs during the later reproductive stages. NCE showed the most consistent numerical advantage at R7 across the three years. Nevertheless, because the significant treatment × growth stage interaction demonstrates that treatment effects depended on growth stage, treatment performance should be interpreted separately at each growth stage rather than summarized as a uniform effect throughout reproductive development.

### 2.5. Effects of Treatments on Phytohormone Content

**Figure 4:**
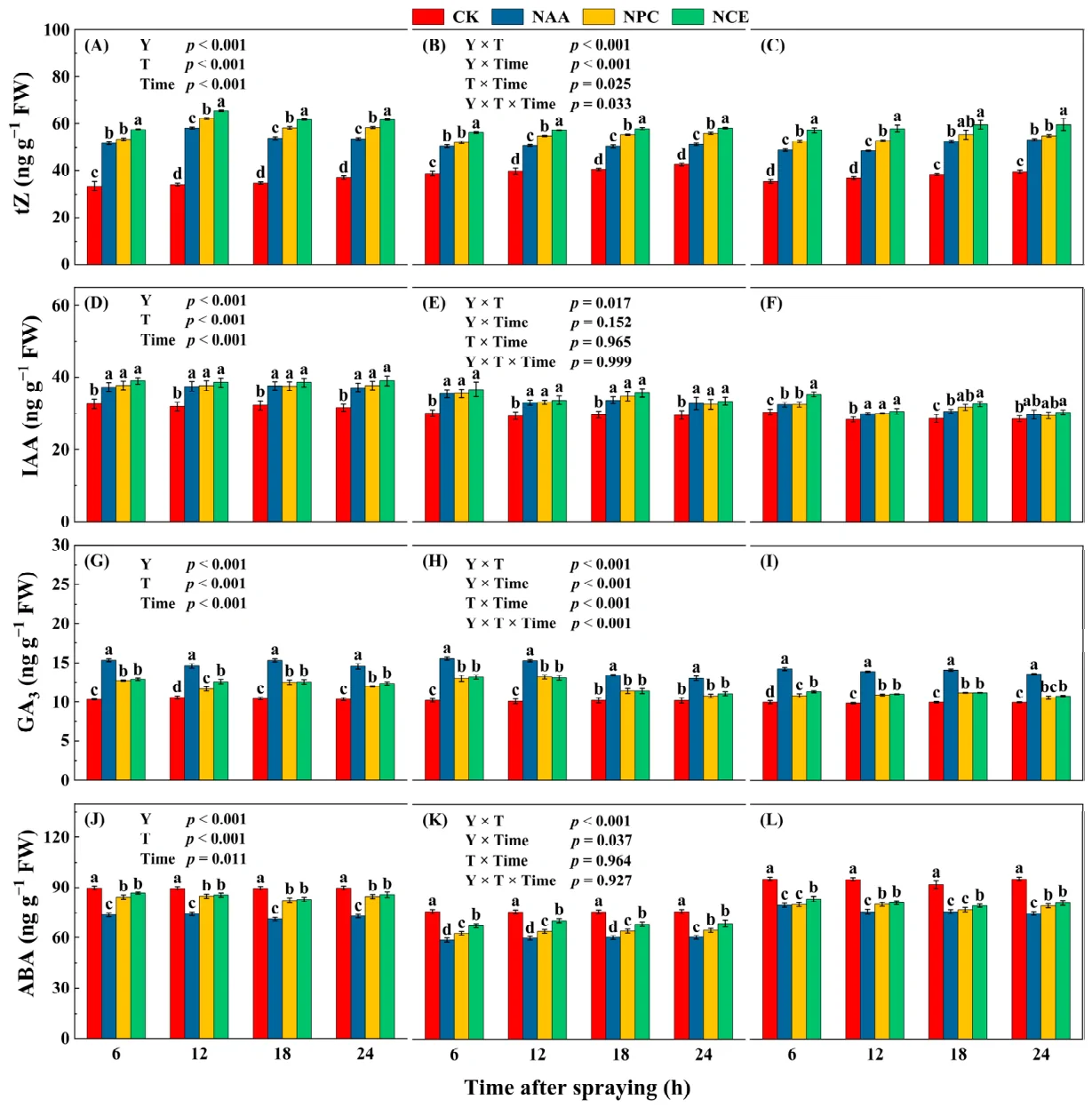
Effects of foliar application of plant growth regulators on leaf phytohormone concentrations in soybeans at the full pod (R4) stage, measured within 24 h after treatment, over three consecutive growing seasons (2023, 2024, and 2025; data in each panel are presented from left to right in chronological order). Hormones measured include tZ (**A**–**C**), IAA (**D**–**F**), GA3 (**G**–**I**), and ABA (**J**–**L**). Error bars represent the standard error of the mean. Different lowercase letters in the vertical direction indicate significant differences between treatments in the same year (*p* < 0.05).

The effects of the foliar treatments on phytohormone contents were generally consistent in direction across the three years: all treatments increased the contents of trans-zeatin (tZ), indole-3-acetic acid (IAA), and gibberellic acid (GA_3_), while decreasing that of abscisic acid (ABA) (Figure 4).

The plot-level time-course analysis showed significant effects of year, treatment, and time on all four phytohormones (*p* ≤ 0.011). For tZ, all two- and three-way interactions were significant (*p* ≤ 0.033, Figure 4A–C). For IAA, only the year × treatment interaction was significant (*p* = 0.017, Figure 4D–F). All interactions were significant for GA_3_ (*p* < 0.001, Figure 4G–I). For ABA, the year × treatment and year × time interactions were significant (*p* < 0.001 and *p* = 0.037, respectively), whereas the other interactions were not significant (*p* ≥ 0.927, Figure 4J–L).

NAA, NPC, and NCE significantly increased tZ content at 6–24 h after application. Relative to CK, tZ content increased by 20.6–70.5%, 31.5–81.9%, and 37.1–91.9% under NAA, NPC, and NCE, respectively. NCE generally produced the greatest increase and resulted in significantly higher tZ contents than NAA and NPC at most sampling times. However, NCE and NPC did not differ significantly at 18 h in 2025.

All treatments also increased IAA content, although the differences among treatments were less pronounced than those observed for tZ. Relative to CK, IAA content increased by 4.2–17.7%, 3.3–19.0%, and 6.2–23.2% under NAA, NPC, and NCE, respectively. In 2023 and 2024, all treatments significantly increased IAA content at 6–24 h, with no significant differences among the three treatments. In 2025, all treatments significantly increased IAA content at 6 and 12 h. At 18 h, significant increases were observed under NAA and NCE, whereas at 24 h, only NCE differed significantly from CK.

NAA exerted the strongest stimulatory effect on GA_3_ content. Relative to CK, NAA increased GA_3_ content by 28.3–52.7% and produced significantly higher values than the other treatments at all sampling times. NPC and NCE increased GA_3_ content by 5.8–31.3% and 8.1–30.2%, respectively. Both treatments significantly increased GA_3_ content at all sampling times in 2023 and at 6–18 h in 2025. In 2024, neither NPC nor NCE differed significantly from CK at 18 or 24 h. At 24 h in 2025, only NCE remained significantly higher than CK.

All treatments reduced ABA content. Relative to CK, ABA content decreased by 16.6–22.6%, 5.3–17.1%, and 3.6–15.1% under NAA, NPC, and NCE, respectively. NAA generally produced the lowest ABA content and differed significantly from NPC and NCE at most sampling times. However, NAA and NPC did not differ significantly at 6 or 18 h in 2025.

### 2.6. Correlation Analysis

**Figure 5:**
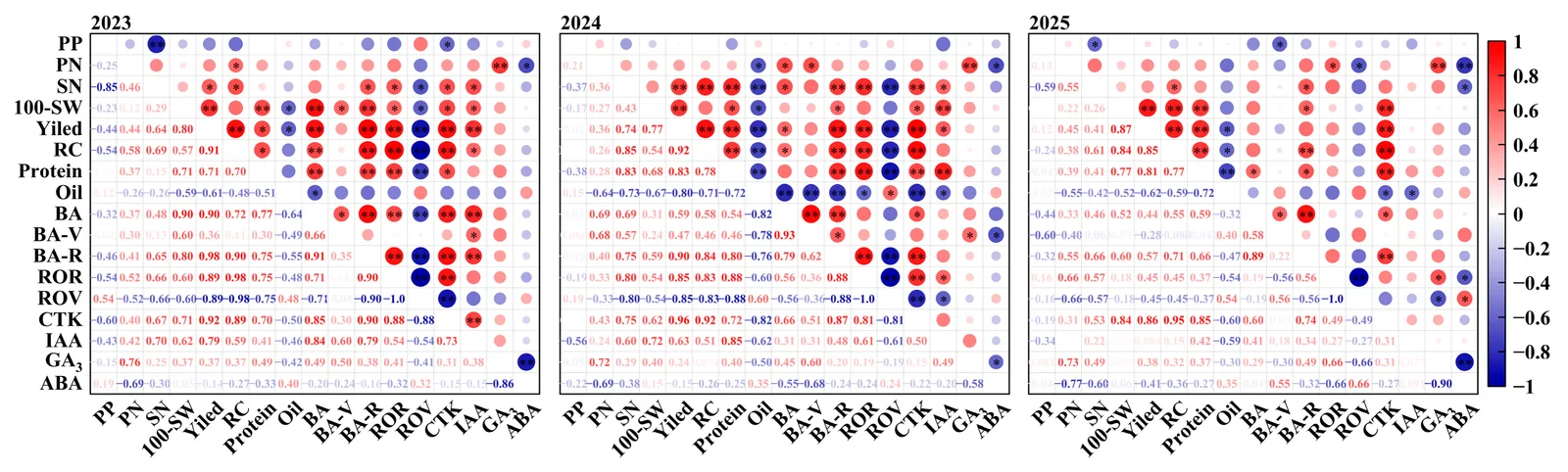
Pearson correlation matrix among soybean yield, total above-ground biomass accumulation (BA), vegetative organ biomass (BA-V), reproductive organ biomass (BA-R), reproductive organ allocation ratio (ROR), vegetative organ allocation ratio (ROV), grain residue (RC), grain quality traits (protein and oil contents), phytohormones (tZ, IAA, GA_3_, ABA), plant population density (PP), and yield components (PN, SN, 100-SW). BA denotes total above-ground biomass accumulation; BA-V indicates vegetative organ biomass accumulation; BA-R represents reproductive organ biomass accumulation; ROR refers to reproductive organ allocation ratio; ROV indicates vegetative organ allocation ratio; RC represents grain residue; PP denotes plant population density; PN indicates pod number per plant; SN represents seed number per plant; 100-SW refers to hundred-seed weight. Positive correlations are shown in red and negative correlations in blue, with circle size proportional to the absolute value of r. Asterisks indicate statistical significance (* *p* < 0.05; ** *p* < 0.01).

Correlation analysis identified stable relationships between yield and several key traits across the three growing seasons (Figure 5). Yield showed positive correlations with 100-SW in each year. The correlation coefficients were 0.80, 0.77, and 0.87 for 2023, 2024, and 2025, respectively. All relationships were significant at *p* < 0.01. Yield also correlated positively with RC, with *r* values of 0.91, 0.92, and 0.85 at *p* < 0.01. Protein content maintained a consistent positive association with yield. The *r* values were 0.71, 0.83, and 0.81. These correlations reached significance at *p* < 0.05 in 2023 and at *p* < 0.01 in both 2024 and 2025. Oil content correlated negatively with yield in all years. The *r* values were −0.61, −0.80, and −0.62, with significance at *p* < 0.05 in 2023 and 2025 and at *p* < 0.01 in 2024. tZ was strongly associated with yield. The *r* values were 0.92, 0.96, and 0.86, and all correlations were significant at *p* < 0.01.

SN and 100-SW contributed more consistently to yield formation than PP or PN. SN correlated positively with RC across years, with *r* values of 0.69, 0.85, and 0.61. SN also showed positive associations with BA-R, with *r* values of 0.65, 0.75, and 0.66. Most of these relationships reached significance. The SN–yield correlation was significant in 2023 and 2024 but not in 2025. PP correlated negatively with SN in 2023 and 2025, with *r* values of −0.85 and −0.59. Most other PP correlations were weak or showed no consistent pattern. PN maintained a positive association with GA_3_, with *r* values of 0.76, 0.72, and 0.73 at *p* < 0.01. PN correlated negatively with ABA in each year. The *r* values were −0.69, −0.69, and −0.77. These relationships were significant at *p* < 0.05 in 2023 and 2024 and at *p* < 0.01 in 2025.

Total above-ground Biomass traits exhibited similar patterns among years. BA correlated positively with BA-V, with *r* values of 0.66, 0.93, and 0.58. BA also correlated with BA-R, with *r* values of 0.91, 0.79, and 0.89. All associations were significant. BA-R showed consistent positive correlations with RC, protein, and tZ. The BA-R–RC coefficients were 0.90, 0.84, and 0.71 at *p* < 0.01. The BA-R–protein coefficients were 0.75, 0.80, and 0.66. The BA-R–tZ coefficients were 0.90, 0.87, and 0.74. These results suggest that total above-ground biomass accumulated in reproductive organs was closely linked to grain filling, protein content, and tZ levels.

ROR and ROV were perfectly negatively correlated in all years, with *r* = −1.00 at *p* < 0.01. ROR correlated positively with yield, RC, BA-R, and tZ in 2023 and 2024. ROV showed correlations in the opposite direction. Most of these relationships lost significance in 2025, indicating greater year-to-year variation in biomass partitioning than in total above-ground biomass. GA_3_ and ABA showed negative correlations across years. The *r* values were −0.86, −0.58, and −0.90. This relationship was significant at *p* < 0.01 in 2023 and 2025 but not in 2024.

## 3. Discussion

### 3.1. Yield Stability and Component Contributions

NAA applied alone or combined with Pro-Ca or with both Pro-Ca and ICE-6 increased soybean yield consistently across three growing seasons in Xinjiang. The NCE treatment showed the most stable yield enhancement, ranging from 13.1% to 14.4% relative to the control. No significant year × treatment interactions were detected for yield or most yield components. This finding indicates that the differences among the tested spray programs were relatively consistent despite environmental variation among growing seasons. This consistency may be relevant to practical adoption because predictable responses are important under variable field conditions [38].

The yield increases were mainly driven by enhanced pod set at the middle nodes from R3 to R5 and increased seed weight [39]. In contrast, total node number and pod set at the upper nodes contributed relatively little to the observed yield responses (Figure 2). NAA alone improved middle-node pod retention modestly, increasing retention by 5.4% to 9.1% across years. This result aligns with auxin’s known role in preventing abscission by enhancing auxin signaling in the abscission zone [15,27]. The NPC spray program showed a greater increase in middle-node pod retention than NAA alone, with increases of 11.8% to 15.3% relative to CK across years. The greater response under NPC may be partly related to the growth-restricting effects previously reported for Pro-Ca, such as reduced internode elongation and changes in canopy architecture [16,17,18]. However, plant architecture and assimilate competition were not directly measured in this study, so this interpretation remains tentative [16,17,18].

NCE produced the heaviest seeds across all three years. NCE produced the greatest 100-seed weight across the three years. This response may be related to improved carbon supply during seed filling [40,41]; however, photosynthetic capacity was not directly measured, and the specific contribution of ICE-6 cannot be separated from the other components in the present design. ICE-6 has been reported to enhance chlorophyll stability and photosynthetic efficiency in other crops [21,22,23]. The reported physiological effects of ICE-6 in other crops may provide a possible explanation for the superior performance of the NCE spray program, although this interpretation requires direct physiological measurements and factorial testing in soybeans.

### 3.2. Pod Formation and Distribution Among Node Positions

Pod number at the R7 stage increased following treatment application, indicating that the regulator programs may have promoted pod formation and/or reduced pod loss before maturity. NPC and NCE consistently increased total pod number across all three years. However, the significant treatment × year interaction indicated that the magnitude of these effects varied among years. Such interannual variation may have been associated with differences in environmental conditions and crop growth, although the specific factors responsible could not be identified from the present study.

Pod number also differed significantly among main-stem node positions, reflecting the inherent spatial distribution of reproductive development along the soybean stem. However, neither the treatment × node position interaction nor the treatment × node position × year interaction was significant. Thus, the results provide no statistical evidence that the treatments affected particular node positions more strongly than others or that the relative treatment responses among node positions varied across years. Descriptive analyses nevertheless showed substantial increases in pod number at nodes 5–9 under NPC and NCE in some years. For example, compared with CK, NCE increased pod number in this region by approximately 43% in 2024 and 60% in 2025. These within-region differences, however, should not be interpreted as evidence that the middle nodes were more responsive to treatment than the lower or upper nodes.

The observed increase in total pod number may be associated with the physiological effects of the individual components of the spray programs. NAA may promote reproductive development and reduce the abortion of developing reproductive structures through auxin-mediated processes [27]. Pro-Ca may affect gibberellin-regulated vegetative growth and thereby alter assimilate allocation between vegetative and reproductive organs [29]. ICE-6 may also contribute to plant physiological performance. However, its independent contribution cannot be determined because photosynthetic rate, canopy carbon assimilation, and related physiological traits were not measured, and neither an ICE-6-only treatment nor an NAA + ICE-6 treatment was included. Further studies are therefore required to distinguish the individual and interactive contributions of these components.

Changes in plant architecture, canopy light conditions, and source–sink relationships may also have contributed to the observed increase in pod number [40]. However, plant height, internode development, canopy light interception, photosynthetic rate, and carbon assimilation were not directly measured; therefore, these potential mechanisms remain hypothetical. Future studies should combine prespecified random or systematic plant sampling with measurements of canopy architecture, within-canopy light distribution, reproductive organ abortion, and carbon assimilation. Such studies would help clarify how these regulatory programs influence pod formation and persistence at different canopy positions.

### 3.3. Total Above-Ground Biomass Dynamics and Reproductive Allocation

Total above-ground biomass accumulation and allocation patterns provide evidence of differences in source–sink relationships among the tested spray programs [42]. Total above-ground biomass differed among years, treatments, and growth stages. The treatment × growth stage interaction was significant. Thus, treatment effects depended on growth stage. The year × growth stage interaction was also significant. This indicates that biomass accumulation patterns varied among years. However, the year × treatment and three-way interactions were not significant. Therefore, there was no evidence that stage-dependent treatment responses differed among years.

All three active spray programs promoted early reproductive organ formation at R1 relative to CK. NPC produced the strongest effect, increasing reproductive organ biomass by 30.5% to 146.6% compared with the control. This early advantage persisted through R3, where all treatments increased reproductive organ biomass by 28.0% to 98.8%. This early reproductive investment was particularly pronounced under NPC. The strong response under NPC may be consistent with the known effects of NAA on reproductive retention and the reported growth-regulating effects of Pro-Ca [16,17]. However, the individual contributions of these compounds cannot be separated with the present treatment structure.

By R5, treatment effects on total above-ground biomass became more apparent. NAA increased total above-ground biomass by 19.3% to 30.9%, driven largely by reproductive organ growth, which increased by 50.9% to 89.7%. Under the conditions of this study, this pattern suggests that the primary effect of NAA was associated with sink establishment rather than source enhancement. This interpretation is supported by the minimal response of vegetative biomass. NPC and NCE showed similar trends, although their relative gains in reproductive biomass at R5 were smaller. Their earlier increases in reproductive organ biomass may have altered the balance among developing sinks, although sink competition was not directly quantified in this study [43,44].

At R7, NCE showed the highest total above-ground biomass across all three years, exceeding the control by 12.4% to 28.2%. This late-season total above-ground biomass advantage was driven almost entirely by reproductive organs, which increased by 29.7% to 49.1% under NCE. Reproductive allocation ratios at R7 ranged from 40.9% to 45.7% under the control but reached 44.7% to 53.3% under treated plants, with NPC and NCE producing higher allocation than NAA. The progressive increase in reproductive allocation from R5 to R7 across all treatments reflects the natural shift from vegetative to reproductive dominance [45]. This shift was particularly consistent under the NCE spray program across years, suggesting that NCE was associated with a relatively favorable reproductive allocation pattern during grain filling.

### 3.4. Hormonal Responses and Their Associations with Yield-Related Traits

The phytohormone profiling data showed distinct hormone response patterns among the four tested spray programs. NCE produced the strongest and most consistent increases in trans-zeatin content. Within 24 h after application, trans-zeatin levels increased by 37.1% to 91.9% compared with the control. At most sampling times, the increase in trans-zeatin content under NCE was significantly greater than that under NAA or NPC. This result indicates that the NCE spray program was associated with a distinct trans-zeatin response pattern. Trans-zeatin promotes cell division, delays senescence, and enhances sink activity [26,31]. These physiological roles may help explain the greater seed weight observed under the NCE spray program, although a causal relationship was not established.

IAA content increased under all treatments, but the differences among treatments were smaller than for trans-zeatin. NAA, NPC, and NCE increased IAA by 4.2% to 17.7%, 3.3% to 19.0%, and 6.2% to 23.2%, respectively. IAA content was lower under NAA alone than under the other two treatments. This response may reflect feedback regulation of auxin metabolism or conjugation of free auxin to maintain hormonal homeostasis [28,31]. Because auxin biosynthesis and conjugation were not directly measured, this explanation remains speculative.

GA_3_ responses differed markedly among treatments. NAA increased the measured GA_3_ concentration by 28.3–52.7% and produced significantly higher concentrations than the other treatments. Although NAA is an auxin rather than a gibberellin, auxin–gibberellin crosstalk is well documented [19,31], and auxin has been reported to promote gibberellin biosynthesis in certain tissues and developmental contexts. Nevertheless, the mechanism underlying the NAA-induced increase in GA_3_ was not directly examined in this study. NPC and NCE produced smaller increases in GA_3_ concentration than NAA. These results indicate that the temporal GA_3_ response patterns under NPC and NCE differed from those under NAA alone. However, because GA_1_, GA_4_, GA_9_, and GA_20_ were not quantified, the GA_3_ data cannot determine whether Pro-Ca inhibited the GA_20_-to-GA_1_ or GA_9_-to-GA_4_ conversion. Further studies quantifying these substrates and bioactive products are required to identify the specific effects of Pro-Ca on gibberellin metabolism in soybean.

All treatments reduced ABA content, with NAA producing the largest decreases of 16.6% to 22.6%. NPC and NCE reduced ABA by only 5.3% to 17.1% and 3.6% to 15.1%, respectively. Under the well-watered conditions of this experiment, lower ABA levels may reduce stress signaling and promote growth [30,46]. The stronger suppression of ABA under NAA treatment alone may contribute to its relatively less stable total above-ground biomass allocation. ABA plays important roles in regulating carbon partitioning and pod retention under environmental stress [30].

The correlation analysis showed strong associations between trans-zeatin levels and several yield-related traits. Trans-zeatin correlated strongly with yield across all years, with correlation coefficients of 0.92, 0.96, and 0.86. Pod numbers were positively correlated with GA_3_ and negatively correlated with ABA in all three years. The corresponding correlation coefficients were consistent throughout the years. These relationships were stable throughout the years. These associations suggest that trans-zeatin status may be a useful indicator of the physiological state associated with yield formation under the tested conditions. However, correlation analysis does not establish causality, and the observed hormone changes may also reflect downstream responses to differences in plant development or total above-ground biomass accumulation.

### 3.5. Seed Quality Responses and Residue Considerations

The treatments altered seed composition in consistent directions across years. NCE increased protein content by 5.6%, 7.9%, and 11.2% in 2023, 2024, and 2025, respectively, representing the strongest and most progressive effect among treatments. Oil content decreased by 2.1% to 5.6% under all treatments compared with the control, with no significant differences among NAA, NPC, and NCE. The inverse relationship between protein and oil content is well established in soybeans. This relationship reflects competition for carbon and nitrogen resources during seed filling [47]. The correlation analysis further supported this trade-off. Protein content was positively correlated with yield in all three years, with coefficients of 0.71, 0.83, and 0.81. In contrast, oil content was negatively correlated with yield, with coefficients of −0.61, −0.80, and −0.62.

The protein increase observed under NCE may reflect multiple physiological processes. Elevated trans-zeatin levels may promote nitrogen remobilization from vegetative tissues to seeds [26,31]. However, nitrogen remobilization was not directly measured, so this possibility requires further verification. This mechanism could explain the strong correlation between trans-zeatin content and reproductive organ biomass observed in the correlation matrix. The progressively greater protein response observed under NCE from 2023 to 2025 may reflect differences in seasonal environmental conditions or treatment responses among years. Because residual effects in soil and plants were not measured, cumulative or adaptive effects cannot be determined. However, residual effects in the soil or plants were not measured in this study. Therefore, this explanation remains speculative.

Seed residue levels remained below 0.05 mg kg^−1^ for all treatments, meeting food safety thresholds [48,49]. NAA produced the lowest residues, likely due to rapid metabolism and conjugation [50]. NPC and NCE resulted in slightly higher residue levels, particularly in 2024 and 2025. Nevertheless, all residue levels remained well within the safety limits. Residue concentration was positively correlated with yield, with coefficients of 0.91, 0.92, and 0.85. This correlation should not be interpreted as evidence that residues themselves increased yield because residue level was confounded with spray-program intensity and may also reflect differences in compound metabolism, persistence, or plant biomass. Instead, treatments that produced stronger physiological effects may also have resulted in greater residue persistence or accumulation [51]. Alternatively, higher-yielding plants with greater biomass may have accumulated proportionally more of the applied compounds [52]. The significant year × treatment interaction for residue levels indicates that environmental conditions influenced residue metabolism or persistence. Nevertheless, this variation did not affect the overall safety profile.

### 3.6. Study Limitations and Future Directions

The treatment structure represents an important limitation of this study. The experiment included CK, NAA alone, NAA + Pro-Ca, and NAA + Pro-Ca + ICE-6, but did not include Pro-Ca alone, ICE-6 alone, NAA + ICE-6, Pro-Ca + ICE-6, or a complete factorial set of treatment combinations. Consequently, the independent main effects of Pro-Ca and ICE-6 and the potential additive or interactive effects among NAA, Pro-Ca, and ICE-6 could not be statistically separated. The findings should therefore be interpreted as comparisons among four specific spray programs rather than as evidence of independent, coordinated, or synergistic effects of the individual compounds.

The plant-sampling procedure also imposed a limitation on the analysis of node-level pod distribution. Fifteen consecutive plants judged to have uniform and representative growth were selected from the central rows of each plot rather than according to a prespecified random or systematic sampling rule. This procedure may have introduced selection bias by underrepresenting short, lodged, sparsely podded, or otherwise treatment-responsive plants. Although the sampled plants were treated as subsamples and the plot was considered the experimental unit, the node-level results should be interpreted with appropriate caution. Future studies should select plants using a prespecified random or systematic procedure, irrespective of plant height, lodging status, pod number, or other phenotypic characteristics.

Several physiological and environmental measurements required to test the proposed mechanisms were not included. Photosynthetic rate, chlorophyll fluorescence, plant architecture, internode development, canopy light interception, and carbon assimilation were not measured directly. Therefore, explanations involving changes in photosynthetic performance, canopy light conditions, assimilate allocation, and source–sink relationships remain hypothetical. Hormone concentrations were also determined at only a limited number of sampling times. More frequent sampling from the early reproductive stages through seed filling would be required to characterize hormone dynamics and their temporal associations with pod formation and abortion more fully. In addition, soil mineral N, including NO_3_^−^–N and NH_4_^+^–N, was not measured immediately before each topdressing application. Consequently, soil N availability at the time of fertilization and its contribution to crop N uptake and treatment responses could not be quantified. Because the four sampling points occurred during the daytime, nighttime, predawn, and the following morning, respectively, elapsed time after treatment was inherently associated with the time of day. Therefore, although the randomized and rapid sampling procedure minimized bias among treatments within each sampling event, possible diurnal effects on phytohormone concentrations could not be completely separated from time-after-application effects. A limitation of this study is that pretreatment phytohormone samples were not collected; thus, baseline equality among the treatment plots could not be directly verified.

The study was conducted under the well-watered and high-fertility conditions typical of irrigated soybean production in Xinjiang. The responses observed under these conditions may not be directly transferable to water-limited, saline, nutrient-deficient, or otherwise stressful environments. Although NPC and NCE increased total pod number in each of the three experimental years, the significant treatment × year interaction indicated that the magnitude of the treatment response varied among years. Additional multi-year and multi-location studies under contrasting environmental and management conditions are therefore needed to evaluate the stability and broader applicability of these spray programs. Longer-term experiments would also be valuable for assessing the consequences of repeated application, including potential effects on soil properties, compound residues, and subsequent crops.

Future studies should employ a complete factorial design comprising all individual compounds, all pairwise combinations, and the three-compound combination. Such a design would permit statistical estimation of the main and interaction effects of NAA, Pro-Ca, and ICE-6. Direct measurements of photosynthesis, chlorophyll fluorescence, plant and canopy architecture, within-canopy light distribution, carbon assimilation, reproductive organ abortion, and N uptake and remobilization would help test the proposed physiological mechanisms. Soil mineral N should also be measured before each topdressing application to quantify soil N supply and its contribution to crop N availability and fertilizer responses. Finally, multi-location trials conducted under contrasting environmental conditions, together with economic analyses, are required to assess the agronomic reliability and practical feasibility of these spray programs.

## 4. Materials and Methods

### 4.1. Field Experiment

#### 4.1.1. Experimental Site

This field study was conducted at the same experimental station in Chabchal County, Ili, Xinjiang Province, China (43°13′ N, 81°09′ E), from 2023 to 2025. However, a different, spatially separate, and non-overlapping field within the station was used in each growing season. The three annual fields were approximately 500 m apart, and no physical subplot was reused across years. Ili is located in the Ili River Valley in the northern Tianshan Mountains and experiences temperate continental and alpine climates. The average nutrient contents within the 0–30 cm soil depth across the three-year study period were 0.074% total N, 100.5 mg kg^−1^ alkaline N, 16.6 mg kg^−1^ available P (P_2_O_5_), and 128.4 mg kg^−1^ available K (K_2_O). Annual variations in soil nutrient levels were minimal, with total N ranging from 0.065% to 0.079%, alkaline N from 88.5 to 107.3 mg kg^−1^, P_2_O_5_ from 15.4 to 17.7 mg kg^−1^, and K_2_O from 118.3 to 139.2 mg kg^−1^. Soil texture was classified as sandy loam with pH 7.8–8.1 and organic matter content of 1.2–1.4%.

Soil samples were collected from each subplot at a depth of 0–30 cm prior to sowing in each year. Five random sampling points were taken per subplot using a soil auger and composited into one representative sample per subplot. Samples were air-dried, ground to pass through a 1 mm sieve, and analyzed for total N by Kjeldahl digestion, alkaline hydrolysable N by alkaline diffusion, available P by the Olsen method, and available K by ammonium acetate extraction using flame photometry.

**Figure 6:**
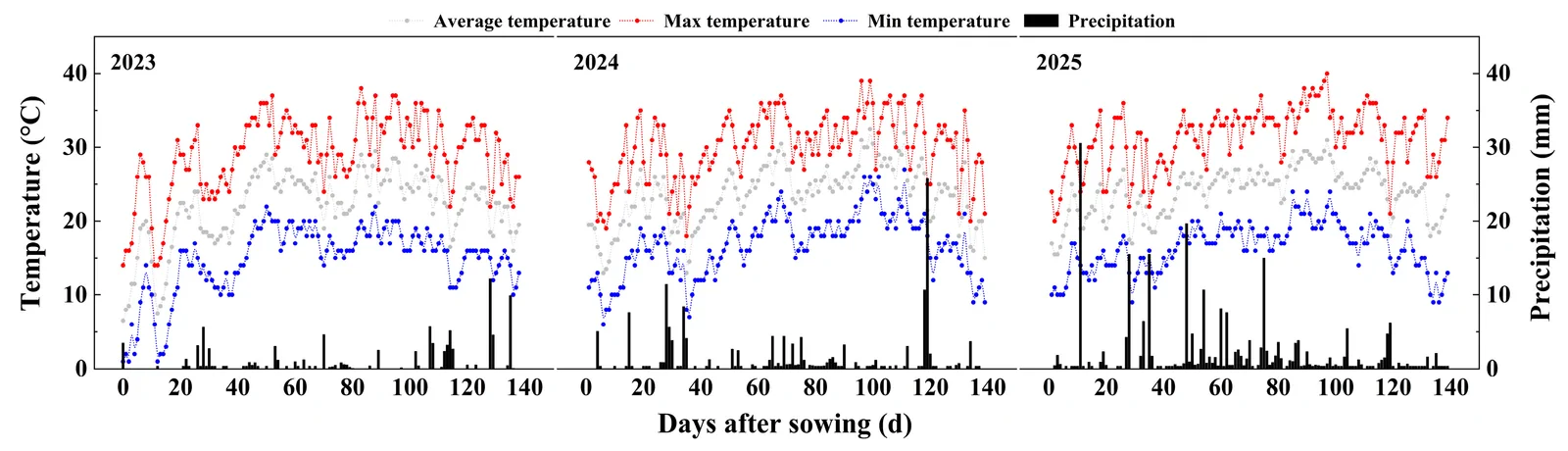
Temperature and precipitation in soybean growing seasons from 2023 to 2025.

Meteorological data during the soybean growing season were obtained from the county agrometeorological station (WMO station class I) located within 2 km of the experimental site. Daily maximum and minimum temperature, precipitation, relative humidity, and sunshine duration were recorded using standard automated weather instruments (HM series automatic weather station, Vaisala Oyj, Helsinki, Finland) following the observation standards of the World Meteorological Organization (WMO-No. 8, 2018) [53]. The weather conditions during the soybean growing season are shown in Figure 6.

#### 4.1.2. Experimental Design and Treatments

In each growing season, a single-factor complete block layout comprising three blocks was used. Each block contained all four treatments once, resulting in three block replicates per treatment and 12 subplots in each annual field. The same complete block layout and treatment arrangement were implemented in each of the three spatially separate annual fields. Thus, the identical treatment arrangements used across years represent the implementation of the same experimental design template in different physical fields rather than repeated use of the same physical subplots.

Four foliar spray treatments were evaluated: (1) NAA treatment applied 5–NAA at 300 g ha^−1^; (2) NPC treatment applied NAA plus 8–Pro-Ca at 300 g ha^−1^ plus 450 g ha^−1^; (3) NCE treatment applied NAA plus 8–Pro-Ca plus 0.01–ICE-6 at 300 g ha^−1^ plus 450 g ha^−1^ plus 45 g ha^−1^; (4) CK treatment applied water as control. All treatments were applied at a spray volume of 450 L ha^−1^ at the V4 and R4 stages using a manually pressurized backpack sprayer. The three annual experiments reported here were conducted in field areas separate from those used in the studies reported in References [13,14], using independent plots, treatment applications, sampling procedures, measurements, and raw data. No experimental units or observations were shared with those previous studies.

Each experimental unit consisted of a 3.6 m × 10 m subplot (36 m^2^) containing eight planted rows spaced 0.45 m apart. The four central rows, each 10 m long, were designated as the net measurement area, resulting in a net measurement area of 18 m^2^ per subplot. The two outer rows on each side served as border rows and were excluded from all plant sampling and measurements. Adjacent subplots were separated by a 2 m-wide interval containing guard rows planted with the same crop. These guard rows received none of the experimental treatments, served solely as untreated buffers, and were excluded from all sampling and measurements.

Treatments were applied over the entire 36-m^2^ subplot using an FST-18DA knapsack electric sprayer (FUSITE Co., Ltd., Taizhou, China) equipped with an 80,015 double conical nozzle. The sprayer was operated at 0.40 MPa and calibrated to deliver 450 L ha^−1^ at a walking speed of 1.0 m s^−1^, corresponding to 1.62 L of spray solution per subplot. Treatments were applied in the order CK, NAA, NPC, and NCE, with CK applied first to minimize the risk of contamination by the plant-growth-regulator treatments. After the application of each plant-growth-regulator treatment, the sprayer tank, spray lance, hose, and nozzle were rinsed five times with clean water before the next treatment solution was prepared. All rinsate was collected and disposed of outside the experimental area.

During treatment application, 2 m-high polyethylene (PE) sheets were temporarily installed along the border rows between adjacent subplots as physical barriers. The PE sheets remained in place throughout the application to each subplot and were removed immediately after that subplot had been sprayed. Together with the 2 m-wide untreated buffer intervals, the treatment application order, and the thorough cleaning of the spraying equipment between treatments, these barriers were used to minimize spray drift and cross-contamination, particularly from the NAA, NPC, and NCE plots into the CK plots. Applications were conducted under dry conditions with no rainfall or visible dew, at an air temperature of 25–29 °C, a relative humidity of 60–75%, and a wind speed of ≤1.5 m s^−1^ at canopy height. No rainfall occurred during application or within 24 h thereafter.

Growth stages were classified according to Fehr and Caviness (1971) [54], where vegetative stages (V1–Vn) are defined by the number of fully developed trifoliate leaves and reproductive stages (R1–R8) represent beginning bloom, full bloom, beginning pod, full pod, beginning seed, full seed, beginning maturity, and full maturity, respectively.

#### 4.1.3. Plant Materials and Field Management

Before the establishment of the annual experiments, the selected fields had been managed under a maize–soybean rotation. All three fields had comparable soil texture and fertility conditions, as described in Section 4.1.1.

The medium-maturity soybean cultivar XND-3 with sub-limited pod formation (Xinjiang Agricultural University, Urumqi, China) was sown on 22 April at 45 cm row spacing and 5 cm plant spacing, resulting in a plant density of approximately 444,000 plants ha^−1^. Seeds of this cultivar are available from Xinjiang Agricultural University upon reasonable request. Three commercial plant growth regulators were used: 5% naphthaleneacetic acid (NAA; Zhengzhou Zhengshi Chemical Co., Ltd., Zhengzhou, China), 8% prohexadione-calcium (Pro-Ca; Chengdu Guanzhi Agricultural Technology Co., Ltd., Chengdu, China), and 0.01% iron chlorin e6 (ICE-6; Nanjing Biotek Biological Engineering Co., Ltd., Nanjing, China). All plant growth regulators and pesticides used in this study are commercially registered products in China. Their application rates and safety intervals complied with the national standard GB/T 8321 (All Parts) and product label instructions. No transgenic materials were involved in this study.

Basal fertilizer (70 kg N, 150 kg P_2_O_5_, 30 kg K_2_O ha^−1^) was incorporated into the soil via rotary tillage before sowing. The basal fertilizer consisted of urea containing 46% N (Shandong Hualu-Hengsheng Group Co., Ltd., Dezhou, China) applied at 152.2 kg ha^−1^, superphosphate containing 18% P_2_O_5_ (Jinan Dasenlin Chemical Technology Co., Ltd., Jinan, China) applied at 833.3 kg ha^−1^, and potassium sulfate containing 52% K_2_O (Handan Yuanwo Fertilizer Technology Co., Ltd., Handan, China) applied at 57.7 kg ha^−1^. An additional 150 kg N ha^−1^ as urea was applied through drip irrigation using the same urea product in three equal split applications. At each of the V3 (third trifoliolate), R1 (beginning bloom), and R3 (beginning pod) stages, urea was applied at 108.7 kg ha^−1^, supplying approximately 50 kg N ha^−1^ per application. Thus, the total N input was approximately 220 kg N ha^−1^. These fertilizer application rates followed conventional local practices for irrigated soybean production in Xinjiang and were applied uniformly across all treatments. Water was supplied via drip irrigation in five events (750 m^3^ ha^−1^ per event) at the V3, R1, R3, R5 (beginning seed), and R6 (full seed) stages, totaling 3750 m^3^ ha^−1^. Soybean seeds were not inoculated with *Bradyrhizobium* before sowing in any experimental season. Weeds were controlled by pre-emergence herbicide application (48% pendimethalin EC at 1.5 L ha^−1^) and manual removal as needed. Pest management followed integrated pest management principles. When the economic thresholds were reached, 4.5% beta-cypermethrin EC (Jiangsu Aijin Crop Technology Group Co., Ltd., Nanjing, China) was applied at a product rate of 225 mL ha^−1^ on 18 July 2023, 26 July 2024, and 22 July 2025. All fertilizer, irrigation, weed-control, and pest-management practices were applied uniformly across treatments within each experimental season, and all other field management practices followed conventional local agricultural standards.

### 4.2. Data Collection

#### 4.2.1. Yield Determination

At the full maturity stage, seed yield was determined by harvesting plants from five randomly located 1 m^2^ quadrats within the central rows of each subplot. The number of harvested plants in each quadrat was recorded. At maturity, all pods borne on the sampled plants, including pods from both the main stem and lateral branches, were collected and counted before threshing. The total number of pods per plant was calculated from the complete pod count, and the plot mean was used for statistical analysis. After pod counting, the plants were threshed, and the seeds were cleaned for subsequent measurements. Pod number per plant was calculated by dividing the total number of pods in each quadrat by the number of harvested plants in the same quadrat. Similarly, seed number per plant was calculated by dividing the total number of seeds in each quadrat by the corresponding number of harvested plants. Seeds were air-dried, and their moisture content was measured using a grain moisture meter (PM-8188-A, Kett Electric Laboratory, Tokyo, Japan). Seed yield was adjusted to a moisture content of 13.5% and expressed as kg ha^−1^ by converting the seed weight harvested from each 1 m^2^ quadrat to a per-hectare basis. The 100-seed weight was determined by randomly selecting and weighing 100 seeds from each quadrat sample in triplicate. The values obtained from the five quadrats were averaged to obtain one value for each subplot for subsequent statistical analyses.

#### 4.2.2. Seed Quality Assessment and Maximum Residue Limit Determination

Seed protein and oil concentrations were quantified according to AOCS protocols. Seeds from individual subplots were pooled, cleaned, and ground using a universal cutting mill (Tecno Dalvo, Santa Fe, Argentina) to pass through a 0.5 mm sieve. Nitrogen content was measured by the Kjeldahl method (Kjeltec 8400, FOSS, Hillerød, Denmark) and converted to protein using a factor of 6.25. Oil was extracted with n-hexane in a Soxhlet apparatus; the solvent was removed by rotary vacuum evaporation at 40 °C, and oil content was determined gravimetrically. All measurements were performed in triplicate.

Based on previous studies, the maximum residue limits (MRLs) of NAA, Pro-Ca, and ICE-6 in soybean seeds were investigated [20,48]. Homogenized seed samples (5.0 g) were extracted with methanol (25 mL) by shaking for 30 min, centrifuged at 4000 rpm for 10 min, and filtered through a 0.45 μm membrane. NAA was quantified via HPLC (Agilent 1260, Agilent Technologies, Santa Clara, CA, USA) on a C18 reversed-phase column (250 mm × 4.6 mm, 5 μm) using methanol–water (70:30, *v*/*v*) at 1.0 mL min^−1^, detection at 280 nm, and external standard calibration. Pro-Ca was analyzed by HPLC with methanol–water (60:40, *v*/*v*) at 1.0 mL min^−1^, detection at 230 nm, and 20 μL injection volume. ICE-6 was quantified by UV–Vis spectrophotometry (UV-2600, Shimadzu, Kyoto, Japan) at 410 nm (Soret band) using external standards. Method validation included linearity (*R*^2^ > 0.999), precision (RSD < 5%), and recovery (85–110%). The limits of detection (LOD) and quantification (LOQ) were 0.001 and 0.003 mg kg^−1^ for NAA, 0.002 and 0.005 mg kg^−1^ for Pro-Ca, and 0.001 and 0.003 mg kg^−1^ for ICE-6, respectively. In accordance with the current Chinese regulatory standard, the general MRL for plant growth regulators is 0.05 mg kg^−1^. No separate commodity-specific MRLs have been established for NAA, Pro-Ca, or ICE-6 [49].

#### 4.2.3. Pod Distribution Measurement

At the R7 stage (beginning maturity), 15 consecutive plants judged to have uniform and representative growth were selected from the central rows of each plot at approximately 11:00 a.m., and the number of pods at each main-stem node was recorded for each plant. The 15 plants were treated as subsamples rather than independent experimental replicates. For plot-level summaries, measurements from the 15 plants were averaged to obtain a single value for each plot, and the plot mean was used as the experimental unit. Each treatment consisted of three independent plot replicates in each year. For the analysis of node-level pod distribution, treatment, node position, year, and all corresponding two- and three-way interactions were included as fixed effects, whereas plot and plant nested within plot were included as random effects to account for the experimental design and the repeated observations made at different nodes of the same plant. Representative plants were photographed for documentation purposes only.

#### 4.2.4. Total Above-Ground Biomass Partitioning

Soybean plants covering a 1.0 m^2^ area were sampled from the non-border rows of each subplot at 11:00 a.m. at the R1, R3, R5, and R7 stages. One sample was collected from each subplot, resulting in three biological replicates per treatment at each growth stage. Plant samples were dissected into vegetative organs (stems, leaves, and petioles) and reproductive organs (flowers, pods, and seeds), excluding abscised material. At the R1 stage, reproductive dry mass comprised retained flower buds, open flowers, and any visible young reproductive structures present on the sampled plants at harvest. Tissues were oven-dried at 105 °C for 30 min for enzyme deactivation, followed by drying at 80 °C to constant weight. Dry mass was determined using an electronic balance (AUW220D, Shimadzu, Kyoto, Japan) with 0.01 g precision. Total above-ground biomass was calculated as the sum of the dry mass of all sampled vegetative and reproductive organs. Biomass allocation to reproductive organs was calculated as the ratio of reproductive organ dry mass to total above-ground biomass. Three independent subsamples were measured within each subplot, and their mean was used as the subplot-level observation for statistical analysis.

#### 4.2.5. Phytohormone Extraction and Quantification

Foliar application began at approximately 10:00 at the R4 growth stage. Fully expanded and healthy leaves from the middle of the inverted third node were collected from soybean plants in each treatment subplot at 16:00–16:20, 22:00–22:20, 04:00–04:20 the following day, and 10:00–10:20 the following day, corresponding to approximately 6, 12, 18, and 24 h after application, respectively. At each sampling event, multiple researchers simultaneously collected samples from the plots in a randomized order, allowing all samples to be collected within 20 min. The samples were immediately wrapped in aluminum foil, flash-frozen in liquid nitrogen, and stored at −80 °C until analysis. Although plot sampling was randomized, the exact plot-by-plot sampling sequence was not retained in the experimental records.

Three independent biological replicates were collected for each treatment and sampling time, with each replicate consisting of pooled leaf material from three plants within the same subplot.

Trans-zeatin (tZ), indole-3-acetic acid (IAA), abscisic acid (ABA), and gibberellic acid (GA_3_) were quantified using an Agilent 1290 high-performance liquid chromatography system coupled to an Agilent 6495 triple-quadrupole mass spectrometer equipped with an electrospray ionization source (Agilent Technologies, Santa Clara, CA, USA). Frozen leaf tissue (0.5 g) was ground in liquid nitrogen. Before extraction, d_5_-zeatin, [^2^H_5_]-IAA, [^2^H_6_]-ABA, and [^2^H_2_]-GA_3_ were added as internal standards at 10 ng per sample to compensate for extraction losses, matrix effects, and variations in instrumental response.

Samples were extracted with 5 mL of n-propanol/water/HCl (2:1:0.002, *v*/*v*/*v*) at 4 °C for 30 min with shaking at 100 rpm. Dichloromethane (2 mL) was then added, followed by shaking for an additional 30 min. After centrifugation at 13,000 rpm for 5 min at 4 °C, 2 mL of the organic phase was collected, frozen at −80 °C for 14 h, and lyophilized for 24 h. The dried extract was reconstituted in 200 μL of 50% (*v*/*v*) methanol, vortex-mixed, and filtered through a 0.22-μm membrane before analysis [55].

Chromatographic separation was performed on an Eclipse Plus C18 column (2.1 × 50 mm, 1.8 μm; Agilent Technologies) maintained at 35 °C. Mobile phase A was water containing 0.1% (*v*/*v*) formic acid, and mobile phase B was methanol containing 0.1% (*v*/*v*) formic acid. The flow rate was 0.3 mL min^−1^, and the injection volume was 2 μL. The gradient program was as follows: 0–1 min, 20% B; 1–8 min, 20–80% B; 8–9 min, 80% B; 9–9.5 min, 80–20% B; and 9.5–12 min, 20% B.

Mass-spectrometric data were acquired in the multiple-reaction-monitoring mode with polarity switching. Trans-zeatin and d_5_-zeatin were detected in the positive-ion mode, whereas IAA, ABA, GA_3_, and their corresponding isotope-labeled internal standards were detected in the negative-ion mode. Compounds were identified by comparing their retention times and MRM transitions with those of authentic standards. The compound-specific MRM transitions, collision energies, fragmentor voltages, retention times, and ion-source parameters are provided in Supplementary Tables S1 and S2.

Quantification was performed using analyte-to-internal-standard peak-area ratios and internal-standard calibration curves prepared from authentic standards. Phytohormone concentrations were expressed as ng g^−1^ fresh weight (FW). The limits of detection (LOD) and quantification (LOQ) were defined as the lowest concentrations producing signal-to-noise ratios of at least 3 and 10, respectively. The LOD and LOQ were 0.5 and 1.5 ng g^−1^ FW for tZ, 1.0 and 3.0 ng g^−1^ FW for IAA, 2.0 and 5.0 ng g^−1^ FW for ABA, and 0.8 and 2.5 ng g^−1^ FW for GA_3_, respectively.

Method validation was performed using blank or low-background soybean leaf matrices fortified at the lower limit of quantification and at low, medium, and high quality-control levels. Extraction recovery, matrix effects, accuracy, and intra- and inter-day precision were evaluated according to the procedures described in the Supplementary Methods. Accuracy and precision were required to be within 85–115% of the nominal concentration and ≤15% RSD, respectively; at the lower limit of quantification, the corresponding acceptance criteria were 80–120% and ≤20% RSD. Detailed calculation formulas and validation results are provided in the Supplementary Methods and Supplementary Table S3.

### 4.3. Statistical Analysis

Data were analyzed using IBM SPSS Statistics 26.0 (IBM Corp., Armonk, NY, USA). The subplot was considered the experimental unit. Measurements from plants, subsamples, or technical replicates within the same subplot were averaged, or summed where appropriate, to obtain one subplot-level value for each response variable before statistical analysis. Subsamples were not treated as independent experimental replicates. For variables measured at multiple growth stages or post-application sampling times, all prespecified measurements were included in the corresponding analyses rather than only selecting the final measurement. Phytohormone concentrations measured at approximately 6, 12, 18, and 24 h after foliar application were analyzed as a post-application time course. Biomass-partitioning variables measured at different stages throughout the growing season were analyzed according to their prespecified sampling stages. Pod numbers at individual main-stem nodes were summed to obtain one total pod-number value per subplot; therefore, node position was not included as a factor in the statistical model.

Normality and homogeneity of variance were assessed using the Shapiro–Wilk test and Levene’s test, respectively, together with graphical inspection of model residuals. When necessary, response variables were appropriately transformed to satisfy model assumptions. Statistical inferences for transformed variables were based on the transformed data, whereas untransformed means are presented for ease of interpretation.

For phytohormone variables, a repeated-measures linear mixed-effects model was fitted at the subplot level. Year, treatment, sampling time, and all corresponding interactions were included as fixed effects:

$$Y_{ijkl}=\mu +Y_{i}+T_{j}+H_{l}+{\left(Y\times T\right)}_{ij}+{\left(Y\times H\right)}_{il}+{\left(T\times H\right)}_{jl}+{\left(Y\times T\times H\right)}_{ijl}+B_{k(i)}+\varepsilon_{ijkl}$$

where $Y_{ijkl}$ is the subplot-level observation, μ is the overall mean, $Y_{i}$ is the fixed effect of year, $T_{j}$ is the fixed effect of treatment, $H_{l}$ is the fixed effect of post-application sampling time, $B_{k(i)}$ is the random effect of block nested within year, and $\varepsilon_{ijk}$ is the subplot-level residual error. Sampling time was treated as a categorical fixed factor with four levels: approximately 6, 12, 18, and 24 h after application. Subplot was specified as the subject for repeated observations. A first-order autoregressive AR(1) covariance structure was used to model the residual correlations among measurements obtained from the same subplot because the sampling times were equally spaced and the correlations were expected to decrease with increasing temporal separation. The random block effect and residual error were assumed to be independent and normally distributed with means of zero and their respective variances.

For total above-ground biomass-partitioning variables measured at multiple stages throughout the growing season, growth stage was included as a fixed effect together with its interactions with year and treatment, where applicable. The corresponding model was:

$$Y_{ijkl}=\mu +Y_{i}+T_{j}+S_{l}+{\left(Y\times T\right)}_{ij}+{\left(Y\times S\right)}_{il}+{\left(T\times S\right)}_{jl}+{\left(Y\times T\times S\right)}_{ijl}+B_{k(i)}+\varepsilon_{ijkl}$$

where $S_{l}$ represents the prespecified growth stage. Growth stage was treated as a categorical fixed factor, and subplot was specified as the subject for repeated observations across growth stages. A compound-symmetry covariance structure was used to model the residual correlations among subplot-level observations obtained at different growth stages. If total above-ground biomass-partitioning variables were analyzed separately at each stage rather than using a repeated-measures model, replace this paragraph with the exact stage-specific analysis actually conducted.

For response variables measured at only one prespecified stage or sampling time, the following linear mixed-effects model was used:

$$Y_{ijk}=\mu +Y_{i}+T_{j}+{\left(Y\times T\right)}_{ij}+B_{k(i)}+\varepsilon_{ijk}$$

where the terms are defined as above. Variance components were estimated using restricted maximum likelihood (REML), and denominator degrees of freedom for tests of fixed effects were calculated using the Satterthwaite approximation. A variance-components covariance structure was specified for the random block effect.

Different, spatially separate, and non-overlapping fields were used during the three growing seasons, and new blocks and subplots were established annually. No physical subplot was reused across years. Consequently, the block was nested within year, and the subplot was not treated as a repeated-measures subject across years. For the phytohormone time-course analysis and the analyses of biomass-partitioning variables measured across growth stages, repeated observations only referred to measurements collected from the same subplot within a given year.

When a significant year × treatment × sampling-time interaction was detected for phytohormone variables (*p* < 0.05), treatment effects were examined separately within each year and sampling time. When the three-way interaction was not significant, but the treatment × sampling-time interaction was significant, treatment effects were examined separately at each sampling time, pooling across years where appropriate. Significant year × treatment interactions for variables measured at a single stage or time were examined separately within each year. For variables measured across growth stages, significant treatment × growth-stage or year × treatment × growth-stage interactions were examined at the corresponding growth stages.

When the relevant interaction was not significant, treatment means were compared across the appropriate levels of the remaining factors. Pairwise comparisons among model-estimated marginal means were performed using the Sidak adjustment for multiple comparisons. Statistical significance was declared at *p* < 0.05.

Results from the time-course and growth-stage analyses are reported as model-estimated marginal means ± standard error (SE), together with the tests of the relevant main effects and interaction terms. Error bars in the figures represent SE.

The data were subjected to analysis of variance (ANOVA), followed by Duncan’s multiple range test to determine significant differences among treatment means at the *p* < 0.05 significance level. Different lowercase letters indicate significant differences among treatment means, whereas means sharing the same lowercase letter are not significantly different.

Pearson correlation coefficients were calculated using subplot-level values to examine relationships among seed yield, yield components, biomass-partitioning variables measured throughout the growing season, and phytohormone concentrations measured at 24 h after treatment application. Figures were prepared using Origin 2025 (OriginLab Corporation, Northampton, MA, USA) and Microsoft PowerPoint 2021.

## 5. Conclusions

This three-year field study showed that the four tested spray programs produced different agronomic, physiological, hormonal, and seed-quality responses in soybeans under the well-watered and high-fertility conditions of Xinjiang’s irrigated production systems. Among the tested programs, NCE, consisting of naphthaleneacetic acid, prohexadione-calcium, and iron chlorin e6, produced the greatest and most consistent yield increase relative to CK, ranging from 13.1% to 14.4% across the three growing seasons. This yield advantage was mainly associated with greater middle-node pod retention, higher 100-seed weight, and increased reproductive biomass allocation at maturity.

The NCE spray program was also associated with higher leaf trans-zeatin content shortly after application, greater reproductive biomass allocation, and an increase in seed protein concentration of up to 11.2%. The strong positive associations between trans-zeatin content, reproductive biomass allocation, and grain yield suggest that trans-zeatin status may be a useful indicator of the physiological processes associated with yield formation under the tested conditions. However, these correlations do not establish a causal relationship. Residue concentrations of the applied compounds in harvested seeds remained below 0.05 mg kg^−1^ under the conditions of this study and were below the relevant safety thresholds.

Overall, the results indicate that the NCE spray program was the best-performing of the four tested programs for improving soybean yield and seed protein concentration under Xinjiang’s irrigated production conditions. However, because the experiment did not include Pro-Ca alone, ICE-6 alone, the corresponding pairwise combinations, or a complete factorial treatment set, the independent contributions and possible additive, synergistic, or interaction effects of the three compounds could not be statistically separated. Therefore, the observed responses should be interpreted as effects associated with the tested spray programs rather than as evidence of confirmed synergistic or coordinated action among NAA, Pro-Ca, and ICE-6. Complete factorial experiments, multi-location trials, direct physiological measurements, and economic analyses are needed to verify the underlying mechanisms and practical applicability of these spray programs under contrasting agroecological conditions.

## Figures and Tables

**Table 1:** Effects of PGR treatments on soybean yield and yield components across three growing seasons (2023–2025).

Year	Treatment	Plant Population (10^4^ Plants ha^−1^)	Total Pod Number (per Plant)	Seed Number (per Plant)	100-Seed Weight (g)	Yield (kg ha^−1^)
2023	CK	42.8 a	47.5 b	57.5 c	18.52 b	4546.17 c
	NAA	42.1 a	52.8 a	62.8 b	18.60 b	4910.12 b
	NPC	41.2 a	51.2 a	66.1 a	18.62 b	5060.99 a
	NCE	41.3 a	51.2 a	64.5 ab	19.32 a	5143.33 a
2024	CK	42.0 a	42.8 b	64.1 b	18.58 c	4997.55 c
	NAA	41.9 a	56.5 a	69.8 a	18.67 bc	5448.64 b
	NPC	42.1 a	51.1 a	70.6 a	18.79 b	5579.67 b
	NCE	42.1 a	54.5 a	70.7 a	19.24 a	5714.73 a
2025	CK	42.6 a	43.4 b	61.4 c	18.38 b	4792.14 c
	NAA	42.4 a	51.4 a	67.8 ab	18.45 b	5300.19 b
	NPC	42.0 a	51.6 a	68.9 a	18.71 b	5393.64 ab
	NCE	42.3 a	48.8 a	66.4 b	19.55 a	5479.62 a
Source of variance						
Y		ns	ns	**	ns	**
T		ns	**	**	**	**
Y × T		ns	ns	ns	ns	ns

Note: Total pod number per plant: main stem + lateral branches. For a given trait, treatments with the same lowercase letter within a year were not significantly different, whereas treatments with different lowercase letters differed significantly, based on Duncan’s multiple range test at *p* < 0.05, using a general linear model. ** represents a significant difference at the 1% level; ns represents no significant difference at the 5% level.

**Table 2:** Planned contrasts, effect sizes, and 95% confidence intervals for yield responses to foliar treatments across three years.

Year	Planned Contrast	Effect Estimate (kg ha^−1^)	Relative Difference (%)	95% CI (kg ha^−1^)
2023	NCE–NPC	82.34	1.63	−150.41, 315.09
	NPC–NAA	150.87	3.07	−11.67, 313.41
	NAA–CK	363.92	8.00	303.73, 424.11
	NPC–CK	514.79	11.32	366.30, 663.29
	NCE–CK	597.13	13.13	477.25, 717.01
2024	NCE–NPC	135.06	2.42	11.56, 258.56
	NPC–NAA	131.03	2.40	−44.11, 306.17
	NAA–CK	451.09	9.03	331.56, 570.62
	NPC–CK	582.12	11.65	460.72, 703.52
	NCE–CK	717.18	14.35	706.16, 728.20
2025	NCE–NPC	85.98	1.59	−313.98, 485.94
	NPC–NAA	93.45	1.76	−259.30, 446.19
	NAA–CK	508.05	10.60	370.93, 645.19
	NPC–CK	601.50	12.55	382.40, 820.60
	NCE–CK	687.48	14.35	436.70, 938.26

Note: Effect estimates were calculated as the mean yield of the first treatment minus that of the second treatment. Relative differences were calculated using the second treatment as the denominator. Positive estimates indicate a higher yield for the first treatment. Confidence intervals containing zero indicate that the corresponding contrast was not statistically significant. The prespecified contrasts were NCE vs. NPC, NPC vs. NAA, and each treatment vs. CK. Confidence intervals were calculated from the three corresponding replicate-level paired differences within each year.

## Data Availability

The data presented in this study are available on request from the corresponding author. Data is contained within the article or Supplementary Material.

## Supplementary Materials

The following supporting information can be downloaded at https://www.mdpi.com/article/10.3390/plants15162528/s1. Table S1. Compound-specific MRM parameters for phytohormone analysis; Table S2. Chromatographic and mass-spectrometric conditions; Table S3. Sensitivity and method-validation results for phytohormone quantification in soybean leaves; Methods: statistical analysis using linear mixed-effects models.

## Abbreviations

The following abbreviations are used in this manuscript:

NAA	Naphthaleneacetic acid
Pro-Ca	Prohexadone-calcium
ICE-6	Iron chlorin e6
NPC	NAA + Pro-Ca combined treatment
NCE	NAA + Pro-Ca + ICE-6 combined treatment
CK	Control (untreated)
V4	Fourth trifoliolate stage
R1	Beginning flower stage
R3	Beginning pod stage
R4	Full pod stage
R5	Beginning seed stage
R7	Beginning maturity stage
tZ	Trans-zeatin
IAA	Indole-3-acetic acid
ABA	Abscisic acid
GA_3_	Gibberellin A3

## References

[1] Waqar A., Li S., Liu Y., Jiang K., Wang S., Yang M., Yuan S., Chen Q., Hu L., Xu L. (2025). Genetic yield improvements and associated physiological and agronomic traits in soybean cultivars released from 1940 to 2021 in Northeast China. Field Crop. Res..

[2] WNBS, PRC Website of National Bureau of Statistics of the People’s Republic of China. https://data.stats.gov.cn/dg/website/page.html#/pc/national/en/yearData.

[3] Nasir B., Rahman S., Ali A., Shafique E., Zia N., Ahmad N., Raza G., Bukhari R. (2025). Resilient soybeans for a changing climate: Analyzing traditional and emerging new plant breeding technologies to combat abiotic stresses. Acta Physiol. Plant..

[4] David S., Cesar A., Iker A. (2022). Additive effects of heatwave and water stresses on soybean seed yield is caused by impaired carbon assimilation at pod formation but not at flowering. Plant Sci..

[5] Fang C., Du H., Wang L., Liu B., Kong F. (2024). Mechanisms underlying key agronomic traits and implications for molecular breeding in soybean. J. Genet. Genom..

[6] Wang X., Gao S., Yang H., Li E., Xie R., Wang K., Zhang G., Yu H., Ming B., Li S. (2026). Spatiotemporal dynamics of maize planting density in China: Yield responses and region-specific intensification thresholds. Crop Environ..

[7] Vymyslický T., Trněný O., Rietman H., Balko C., Đorđević V., Ranđelović P., Dybová M. (2025). Phenotypic characterization of soybean genetic resources at multiple locations: Breeding implications for enhancing environmental resilience, yield and protein content. Front. Plant Sci..

[8] Ahmad I., Zhu G., Zhou G., Younas M., Yan X. (2025). Melatonin and mepiquat chloride impact on cotton growth, physiological and yield at different growth stages. Ind. Crops Prod..

[9] Sreekanth D., Singh C., Yadav M., Singh T., Kuwardadra S., Pawar D. (2026). Phytohormones and plant growth regulators as sustainable modulators of salinity stress tolerance in major crops: Mechanistic insights and environmental perspectives. Plant Stress.

[10] Mabotja M., Aremu A., Doležal K., Ruzvidzo O., Amoo S. (2025). Enhancing in vitro propagation of *Melissa officinalis* L.: Assessing the role of plant growth regulators as a pathway to sustainable production. Ind. Crops Prod..

[11] Razzaq S., Zhou B. (2025). Revolutionizing crop production with iron nanoparticles for controlled release of plant growth regulators and abiotic stress resistance. Plant Nano Biol..

[12] Zhong X., Wu Q., Yang L., Lin Z., Ren D., Li Z., Zhou Y., Duan L. (2026). Brazide: A breakthrough plant growth regulator that delays leaf senescence under heat stress to promote grain yield in winter wheat (*Triticum aestivum* L.). Field Crop. Res..

[13] Cheng H., Xu Q., Ding C., Meng Z., Zhao F., Gan Y., Song X., Zhao Q. (2025). Plant Growth Regulators Reduce Flower and Pod Shedding and Optimize Pod Distribution in Soybean in Northwest China. Agronomy.

[14] Cheng H., Gan Y., Zheng X., Meng Z., Zhao F., Feng W., Guo R., Song X., Zhao Q. (2025). Plant Growth Regulators Improve Soybean Yield in Northwest China Through Nutritional and Hormonal Regulation. Agronomy.

[15] Wu Y., Wu Q., Luo Y., Syaifudin M., Tang X., Li T., Chen J., Du H. (2026). Regulation of vegetative propagation by exogenous α-NAA and GA1 in *Sargassum hemiphyllum* var. *chinense*. Algal Res..

[16] Jayasinghege C., Burlakoti R., Bineng C. (2026). Controlling cranberry canopy growth using the gibberellin biosynthesis inhibitor prohexadione-calcium. Sci. Hortic..

[17] Baró A., DuPont S. (2026). Control of shoot blight in young apple trees of the cultivar ‘WA 38’ under field conditions using prohexadione-calcium and acibenzolar-S-methyl. Physiol. Mol. Plant Pathol..

[18] Feng N., Yu M., Li Y., Jin D., Zheng D. (2021). Prohexadione-calcium alleviates saline-alkali stress in soybean seedlings by improving the photosynthesis and up-regulating antioxidant defense. Ecotoxicol. Environ. Saf..

[19] Ross J., O’Neill D., Wolbang C., Symons G., Reid J. (2014). Auxin-Gibberellin Interactions and Their Role in Plant Growth. J. Plant Growth Regul..

[20] Zhou W., Chen J., Zhou R., Xiao J., Li Y., Ren Y., Li B. (2024). Evaluation of Iron Chlorin e6 disappearance and hydrolysis in soil and garlic using salting-out assisted liquid-liquid extraction coupled with high-performance liquid chromatography and ultraviolet-visible detection. Food Chem..

[21] Yang D., Li Z., He Y., Ma X., Shen J., Xun C., Zhang Z., Wang R., Chen Y., He Z. (2025). Iron Chlorin E6 enhances drought resilience in *Camellia oleifera Abel*. Front. Plant Sci..

[22] Feng Q., Lu L., Li Q., Wang L., Pei Q., Pan S., Tian J., Lu S., Li S. (2024). Effect of a new plant growth regulator iron chlorin 0.02% soluble powder on the growth and yield of grape. Hortic. Sci..

[23] Xie Y., Wei L., Ji Y., Li S. (2022). Seed Treatment with Iron Chlorine E6 Enhances Germination and Seedling Growth of Rice. Agriculture.

[24] Yang L., Chen X., Jin W., Zhou J., Xu Y., Liu R., Song W., Kong L., Huang Z., Du X. (2025). Integrating source-sink coordination and pod-setting optimization: A field study on plant density effects for soybean productivity enhancement in the Huang-Huai-Hai Plain. J. Agric. Food Res..

[25] Yu S., Lo S., Ho T. (2015). Source–sink communication: Regulated by hormone, nutrient, and stress cross-signaling. Trends Plant Sci..

[26] Wu W., Du K., Kang X., Wei H. (2021). The diverse roles of cytokinins in regulating leaf development. Hortic. Res..

[27] Adem M., Sharma L., Shekhawat G., Šafranek M., Jásik J. (2024). Auxin signaling, transport, and regulation during adventitious root formation. Curr. Plant Biol..

[28] Ament A., Kuchařová A., Vladejić J., Bělíček J., Brunoni F., Novák O. (2025). Auxin-mediated seed germination and crosstalk with other phytohormones. Front. Plant Sci..

[29] Shah S., Islam S., Mohammad F., Siddiqui M. (2023). Gibberellic acid: A versatile regulator of plant growth, development and stress responses. J. Plant Growth Regul..

[30] Kishor P., Tiozon R., Fernie A., Sreenivasulu N. (2022). Abscisic acid and its role in the modulation of plant growth, development, and yield stability. Trends Plant Sci..

[31] Roychoudhury A., Aftab T. (2021). Phytohormones, plant growth regulators and signaling molecules: Cross-talk and stress responses. Plant Cell Rep..

[32] Ahmed I., Ali E., Gad A., Bardisi A., El-Tahan A., Abd Esadek O., Gendy A. (2022). Impact of plant growth regulators spray on fruit quantity and quality of pepper (*Capsicum annuum* L.) cultivars grown under plastic tunnels. Saudi J. Biol. Sci..

[33] Dubey A., Malla M., Kumar A., Khan M., Kumari S. (2024). Seed bio-priming with ACC deaminase-producing bacterial strains alleviates impact of drought stress in Soybean (*Glycine max* (L.) Merr.). Rhizosphere.

[34] Musacchi S., Sheick R., Mia M., Serra S. (2023). Studies on physiological and productive effects of multi-leader training systems and Prohexadione-Ca applications on apple cultivar ‘WA 38’. Sci. Hortic..

[35] Huang X., Zheng D., Feng N., Huang A., Zhang R., Meng F., Zhou H. (2023). Effects of prohexadione calcium spraying during the booting stage on panicle traits, yield, and related physiological characteristics of rice under salt stress. PeerJ.

[36] Ishfaq S., Ding Y., Liang X., Guo W. (2025). Advancing lodging resistance in maize: Integrating genetic, hormonal, and agronomic insights for sustainable crop productivity. Plant Stress.

[37] Cokuraj M., Manokari M., Mohammad F., Alatar A., Shekhawat M. (2025). Synergistic effects of growth regulators and silicon nanoparticles on enhancing morpho-structural stability in *Vitex agnus-castus* L. plantlets. S. Afr. J. Bot..

[38] Han G., Niles M. (2023). An adoption spectrum for sustainable agriculture practices: A new framework applied to cover crop adoption. Agric. Syst..

[39] Hou Z., Liu B., Kong F. (2022). Chapter Two—Regulation of flowering and maturation in soybean. Adv. Bot. Res..

[40] Nico M., Mantese A., Miralles D., Kantolic A. (2015). Soybean fruit development and set at the node level under combined photoperiod and radiation conditions. J. Exp. Bot..

[41] Pandit M., Timilsina A., Nzaramyimana T., Upadhaya S., Chiluwal A. (2026). Impact of supplemental nitrogen application at different reproductive stages on soybean yield and seed composition. J. Agric. Food Res..

[42] Tsogtsaikhan T., Yang X., Gao R., Liu J., Tang W., Liu G., Ye X., Huang Z. (2025). Biomass allocation between reproductive and vegetative organs of Artemisia along a large environmental gradient. BMC Plant Biol..

[43] Di Mauro G., Rotundo J. (2025). Lodging dynamics and seed yield for two soybean genotypes with contrasting lodging-susceptibility. Eur. J. Agron..

[44] Vogel J., Liu W., Olhoft P., Crafts-Brandner S., Pennycooke J., Christiansen N. (2021). Soybean Yield Formation Physiology—A Foundation for Precision Breeding Based Improvement. Front. Plant Sci..

[45] Bonser S., Aarssen L. (2009). Interpreting reproductive allometry: Individual strategies of allocation explain size-dependent reproduction in plant populations. Perspect. Plant Ecol..

[46] Teng Z., Lyu J., Chen Y., Zhang J., Ye N. (2023). Effects of stress-induced ABA on root architecture development: Positive and negative actions. Crop J..

[47] Shen S., Ma S., Wu L., Zhou S., Ruan Y. (2023). Winners take all: Competition for carbon resource determines grain fate. Trends Plant Sci..

[48] Kim K., Park D., Kang G., Kim T., Yang Y., Moon S., Choi E., Ha D., Kim E., Cho B. (2016). Simultaneous determination of plant growth regulator and pesticides in bean sprouts by liquid chromatography–tandem mass spectrometry. Food Chem..

[49] (2021). National Food Safety Standard—Maximum Residue Limits for Pesticides in Food.

[50] Musazade E., Mrisho I., Feng X. (2025). Auxin metabolism and signaling: Integrating independent mechanisms and crosstalk in plant abiotic stress responses. Plant Stress.

[51] Hafeez M., Zahra N., Zahra K., Raza A., Batool A., Shaukat K., Khan S. (2021). Brassinosteroids: Molecular and physiological responses in plant growth and abiotic stresses. Plant Stress.

[52] Zhang Z., Shao S., Pan D., Wu X. (2024). Role, residues and microbial degradation of plant growth regulators (PGRs): A scoping review. Adv. Agrochem..

[53] World Meteorological Organization (2018). Guide to Instruments and Methods of Observation (WMO-No. 8).

[54] Fehr W., Caviness C. (1971). Special Report 80, Iowa Agricultural Experiment Station. Stages of Soybean Development.

[55] Pan X., Welti R., Wang X. (2010). Quantitative analysis of major plant hormones in crude plant extracts by high-performance liquid chromatography–mass spectrometry. Nat. Protoc..

