# Supplementary material for: Agronomic, Hormonal, and Seed Quality Responses of Soybeans to Four Spray Treatment Regimes Under Irrigated Conditions in Xinjiang: A Three-Year Field Study
Source: Plants (Basel). 2026 Aug 20;15(16):2528. doi: 10.3390/plants15162528 (PMC13516119; doi:10.3390/plants15162528)
Supplement: Supplementary file 1 [file plants-15-02528-s001.zip › Supplementary Method-Supplementary Methods Statistical Analysis Using Linear Mixed Models.pdf]

All response variables were analyzed using linear mixed models in IBM SPSS Statistics. Year, treatment, time, node, stage, year × treatment, year × node, year × time, year × stage, treatment × time, treatment × stage, treatment × node, year × treatment × node, year × treatment × time, and the year × treatment × stage interaction were included as fixed effects, whereas block nested within year was included as a random effect. Models were fitted using restricted maximum likelihood (REML), and denominator degrees of freedom were calculated using the Satterthwaite approximation. Type III tests were used to evaluate the fixed effects.

Estimated marginal means were calculated for the year × treatment interaction and for the treatment main effect. Treatments were compared separately within each year to evaluate treatment differences under the conditions of a given year. In addition, treatment means averaged across years were compared to evaluate the overall treatment effect across years. Pairwise-comparison P-values were adjusted using the Sidak method.

The year × treatment interaction was considered when interpreting these comparisons. When the interaction was significant, primary emphasis was placed on treatment comparisons within individual years because treatment effects differed among years. When the interaction was not significant, the overall treatment comparisons averaged across years were used to summarize the general treatment effect. Within-year comparisons were also presented where they addressed the prespecified objectives of the study.

Residual distributions and homogeneity of variance were assessed using model diagnostic plots. When necessary, response variables were transformed before analysis. Residual concentrations containing true zero values were transformed as $\ln(x + 1)$, where applicable. Statistical tests for transformed variables were conducted on the transformed scale, whereas descriptive means may be presented on the original scale for ease of interpretation. The significance level was set at $p < 0.05$.

```
* Plant population.
MIXED Plant_population BY year treatment block
  /FIXED = year treatment year*treatment | SSTYPE(3)
  /METHOD = REML
  /RANDOM = INTERCEPT | SUBJECT(year*block) COVTYPE(VC)
  /CRITERIA = DFMETHOD(SATTERTHWAITE)
  /EMMEANS = TABLES(year*treatment) COMPARE(treatment) ADJ(SIDAK)
  /EMMEANS = TABLES(treatment) COMPARE(treatment) ADJ(SIDAK)
  /PRINT = SOLUTION TESTCOV.


* Pod number.
MIXED Pod_number BY year treatment block
  /FIXED = year treatment year*treatment | SSTYPE(3)
  /METHOD = REML
  /RANDOM = INTERCEPT | SUBJECT(year*block) COVTYPE(VC)
  /CRITERIA = DFMETHOD(SATTERTHWAITE)
  /EMMEANS = TABLES(year*treatment) COMPARE(treatment) ADJ(SIDAK)
  /EMMEANS = TABLES(treatment) COMPARE(treatment) ADJ(SIDAK)
  /PRINT = SOLUTION TESTCOV.
```

```
* Seed number.
MIXED Seed_number BY year treatment block
  /FIXED = year treatment year*treatment | SSTYPE(3)
  /METHOD = REML
  /RANDOM = INTERCEPT | SUBJECT(year*block) COVTYPE(VC)
  /CRITERIA = DFMETHOD(SATTERTHWAITE)
  /EMMEANS = TABLES(year*treatment) COMPARE(treatment) ADJ(SIDAK)
  /EMMEANS = TABLES(treatment) COMPARE(treatment) ADJ(SIDAK)
  /PRINT = SOLUTION TESTCOV.


* Seed weight.
MIXED Seed_weight BY year treatment block
  /FIXED = year treatment year*treatment | SSTYPE(3)
  /METHOD = REML
  /RANDOM = INTERCEPT | SUBJECT(year*block) COVTYPE(VC)
  /CRITERIA = DFMETHOD(SATTERTHWAITE)
  /EMMEANS = TABLES(year*treatment) COMPARE(treatment) ADJ(SIDAK)
  /EMMEANS = TABLES(treatment) COMPARE(treatment) ADJ(SIDAK)
  /PRINT = SOLUTION TESTCOV.


* Yield.
MIXED Yield BY year treatment block
  /FIXED = year treatment year*treatment | SSTYPE(3)
  /METHOD = REML
  /RANDOM = INTERCEPT | SUBJECT(year*block) COVTYPE(VC)
  /CRITERIA = DFMETHOD(SATTERTHWAITE)
  /EMMEANS = TABLES(year*treatment) COMPARE(treatment) ADJ(SIDAK)
  /EMMEANS = TABLES(treatment) COMPARE(treatment) ADJ(SIDAK)
  /PRINT = SOLUTION TESTCOV.


* Residual concentrations.
MIXED Residual_concentrations BY year treatment block
  /FIXED = year treatment year*treatment | SSTYPE(3)
  /METHOD = REML
  /RANDOM = INTERCEPT | SUBJECT(year*block) COVTYPE(VC)
  /CRITERIA = DFMETHOD(SATTERTHWAITE)
  /EMMEANS = TABLES(year*treatment) COMPARE(treatment) ADJ(SIDAK)
  /EMMEANS = TABLES(treatment) COMPARE(treatment) ADJ(SIDAK)
  /PRINT = SOLUTION TESTCOV.
```

```
* Protein content.
MIXED Protein_content BY year treatment block
  /FIXED = year treatment year*treatment | SSTYPE(3)
  /METHOD = REML
  /RANDOM = INTERCEPT | SUBJECT(year*block) COVTYPE(VC)
  /CRITERIA = DFMETHOD(SATTERTHWAITE)
  /EMMEANS = TABLES(year*treatment) COMPARE(treatment) ADJ(SIDAK)
  /EMMEANS = TABLES(treatment) COMPARE(treatment) ADJ(SIDAK)
  /PRINT = SOLUTION TESTCOV.


* Oil content.
MIXED Oil_content BY year treatment block
  /FIXED = year treatment year*treatment | SSTYPE(3)
  /METHOD = REML
  /RANDOM = INTERCEPT | SUBJECT(year*block) COVTYPE(VC)
  /CRITERIA = DFMETHOD(SATTERTHWAITE)
  /EMMEANS = TABLES(year*treatment) COMPARE(treatment) ADJ(SIDAK)
  /EMMEANS = TABLES(treatment) COMPARE(treatment) ADJ(SIDAK)
  /PRINT = SOLUTION TESTCOV.


COMPUTE main_stem_node_pods = SUM(pod_n1 TO pod_n15).
EXECUTE.

MIXED main_stem_node_pods BY year treatment block
  /FIXED = year treatment node year*treatment year*node node*treatment
year*treatment*node | SSTYPE(7)
  /METHOD = REML
  /RANDOM = INTERCEPT | SUBJECT(year*block) COVTYPE(VC)
  /CRITERIA = DFMETHOD(SATTERTHWAITE)
  /EMMEANS = TABLES(year*treatment) COMPARE(treatment) ADJ(SIDAK)
  /EMMEANS = TABLES(treatment) COMPARE(treatment) ADJ(SIDAK)
  /PRINT = SOLUTION TESTCOV.


* above ground biomass.
MIXED above_ground_biomass BY year treatment block
  /FIXED = year treatment stage year*treatment year*stage stage*treatment
year*treatment* stage | SSTYPE(7)
  /METHOD = REML
  /RANDOM = INTERCEPT | SUBJECT(year*block) COVTYPE(VC)
```

```
  /CRITERIA = DFMETHOD(SATTERTHWAITE)
  /EMMEANS = TABLES(year*treatment) COMPARE(treatment) ADJ(SIDAK)
  /EMMEANS = TABLES(treatment) COMPARE(treatment) ADJ(SIDAK)
  /PRINT = SOLUTION TESTCOV.


* Reproductive biomass.
MIXED Reproductive_biomass BY year treatment block
  /FIXED = year treatment year*treatment | SSTYPE(3)
  /METHOD = REML
  /RANDOM = INTERCEPT | SUBJECT(year*block) COVTYPE(VC)
  /CRITERIA = DFMETHOD(SATTERTHWAITE)
  /EMMEANS = TABLES(year*treatment) COMPARE(treatment) ADJ(SIDAK)
  /EMMEANS = TABLES(treatment) COMPARE(treatment) ADJ(SIDAK)
  /PRINT = SOLUTION TESTCOV.


* Vegetative biomass.
MIXED Vegetative_biomass BY year treatment block
  /FIXED = year treatment year*treatment | SSTYPE(3)
  /METHOD = REML
  /RANDOM = INTERCEPT | SUBJECT(year*block) COVTYPE(VC)
  /CRITERIA = DFMETHOD(SATTERTHWAITE)
  /EMMEANS = TABLES(year*treatment) COMPARE(treatment) ADJ(SIDAK)
  /EMMEANS = TABLES(treatment) COMPARE(treatment) ADJ(SIDAK)
  /PRINT = SOLUTION TESTCOV.


* Vegetative biomass.
MIXED Vegetative_biomass BY year treatment block
  /FIXED = year treatment year*treatment | SSTYPE(3)
  /METHOD = REML
  /RANDOM = INTERCEPT | SUBJECT(year*block) COVTYPE(VC)
  /CRITERIA = DFMETHOD(SATTERTHWAITE)
  /EMMEANS = TABLES(year*treatment) COMPARE(treatment) ADJ(SIDAK)
  /EMMEANS = TABLES(treatment) COMPARE(treatment) ADJ(SIDAK)
  /PRINT = SOLUTION TESTCOV.


* tZ.
MIXED tZ BY year treatment block
  /FIXED  =  year  treatment  time  year*treatment  year*time  time*treatment
year*treatment*time | SSTYPE(7)
  /METHOD = REML
```

```
  /RANDOM = INTERCEPT | SUBJECT(year*block) COVTYPE(VC)
  /CRITERIA = DFMETHOD(SATTERTHWAITE)
  /EMMEANS = TABLES(year*treatment) COMPARE(treatment) ADJ(SIDAK)
  /EMMEANS = TABLES(treatment) COMPARE(treatment) ADJ(SIDAK)
  /PRINT = SOLUTION TESTCOV.


* IAA.
MIXED IAA BY year treatment block
  /FIXED = year treatment time year*treatment year*time time*treatment
year*treatment*time | SSTYPE(7)
  /METHOD = REML
  /RANDOM = INTERCEPT | SUBJECT(year*block) COVTYPE(VC)
  /CRITERIA = DFMETHOD(SATTERTHWAITE)
  /EMMEANS = TABLES(year*treatment) COMPARE(treatment) ADJ(SIDAK)
  /EMMEANS = TABLES(treatment) COMPARE(treatment) ADJ(SIDAK)
  /PRINT = SOLUTION TESTCOV.


* GA3.
MIXED GA3 BY year treatment block
  /FIXED = year treatment time year*treatment year*time time*treatment
year*treatment*time | SSTYPE(7)
  /METHOD = REML
  /RANDOM = INTERCEPT | SUBJECT(year*block) COVTYPE(VC)
  /CRITERIA = DFMETHOD(SATTERTHWAITE)
  /EMMEANS = TABLES(year*treatment) COMPARE(treatment) ADJ(SIDAK)
  /EMMEANS = TABLES(treatment) COMPARE(treatment) ADJ(SIDAK)
  /PRINT = SOLUTION TESTCOV.


* ABA.
MIXED ABA BY year treatment block
  /FIXED = year treatment time year*treatment year*time time*treatment
year*treatment*time | SSTYPE(7)
  /METHOD = REML
  /RANDOM = INTERCEPT | SUBJECT(year*block) COVTYPE(VC)
  /CRITERIA = DFMETHOD(SATTERTHWAITE)
  /EMMEANS = TABLES(year*treatment) COMPARE(treatment) ADJ(SIDAK)
  /EMMEANS = TABLES(treatment) COMPARE(treatment) ADJ(SIDAK)
  /PRINT = SOLUTION TESTCOV.
```
