# Supplementary material for: Agronomic, Hormonal, and Seed Quality Responses of Soybeans to Four Spray Treatment Regimes Under Irrigated Conditions in Xinjiang: A Three-Year Field Study
Source: Plants (Basel). 2026 Aug 20;15(16):2528. doi: 10.3390/plants15162528 (PMC13516119; doi:10.3390/plants15162528)
Supplement: Supplementary file 1 [file plants-15-02528-s001.zip › Supplementary Tables S1–S3.pdf]

Supplementary Table S1. Compound-specific MRM parameters for phytohormone analysis.

Compound	Abbreviation	Role	Corresponding internal standard	Ionization mode	Precursorion (*m*/*z*)	Production (*m*/*z*)	MRM transition	Fragmentor (V)	Collision energy (eV)	Retention time (min)
Trans-Zeatin	tZ	Analyte	d_5-Zeatin	ESI+	220.1	136.1	220.1→136.1	110	20	2.5
d_5-Zeatin	d_5-tZ	Internal standard for tZ	—	ESI+	225.1	136.1	225.1→136.1	110	20	2.6
Indole-3-acetic acid	IAA	Analyte	[$^2H_5$]-IAA	ESI−	174.1	130.1	174.1→130.1	90	18	3.3
[$^2H_5$]-Indole-3-acetic acid	[$^2H_5$]-IAA	Internal standard for IAA	—	ESI−	179.1	135.1	179.1→135.1	90	18	3.4
Abscisic acid	ABA	Analyte	[$^2H_6$]-ABA	ESI−	263.1	153.1	263.1→153.1	120	25	5.1
[$^2H_6$]-Abscisic acid	[$^2H_6$]-ABA	Internal standard for ABA	—	ESI−	269.1	159.1	269.1→159.1	120	25	5.1
Gibberellic acid	GA_3	Analyte	[$^2H_2$]-GA_3	ESI−	345.1	239.1	345.1→239.1	125	15	4.7
[$^2H_2$]-Gibberellic acid	[$^2H_2$]-GA_3	Internal standard for GA_3	—	ESI−	347.1	241.1	347.1→241.1	125	15	4.7

Table note: ESI+, positive electrospray ionization; ESI−, negative electrospray ionization; MRM, multiple-reaction monitoring. Each stable-isotope-labelled internal standard was added before extraction at 10 ng per sample. Compound identification was based on agreement between the retention time and MRM transition of each analyte and those of the corresponding authentic standard.

Supplementary Table S2. Chromatographic and mass-spectrometric conditions.

(Chromatographic conditions)	
**Parameter**	**Setting**
HPLC system	Agilent 1290 high-performance liquid chromatography system
Analytical column	Eclipse Plus C18 column
Column dimensions	2.1 × 50 mm
Particle size	1.8 μm
Column temperature	35 °C
Mobile phase A	Water containing 0.1% (v/v) formic acid
Mobile phase B	Methanol containing 0.1% (v/v) formic acid
Flow rate	0.3 mL min^{-1}
Injection volume	2 μL
Total run time	12 min

(Gradient-elution program)		
**Time interval (min)**	**Mobile phase B (%)**	**Description**
0–1.0	20	Initial hold
1.1–8.0	20–80	Linear increase
8.1–9.0	80	Hold
9.1–9.5	80–20	Return to initial conditions

9.5–12.0	20	Column re-equilibration

(Ion-source and MS acquisition parameters)	
**Parameter**	**Setting**
Mass spectrometer	Agilent 6495 triple-quadrupole mass spectrometer
Ion source	Electrospray ionization (ESI)
Acquisition mode	MRM with polarity switching
Capillary voltage, positive-ion mode	3500 V
Capillary voltage, negative-ion mode	3000 V
Nozzle voltage	600 V
Drying-gas temperature	250 °C
Drying-gas flow rate	11 L min^{-1}
Nebulizer pressure	35 psi
Sheath-gas temperature	370 °C
Sheath-gas flow rate	10 L min^{-1}
Cell accelerator voltage	5 V
Delta EMV	220 V
Dwell time	30–60 ms

Table note: tZ and d_5-zeatin were monitored in positive-ion mode. IAA, ABA, GA_3, and their corresponding stable-isotope-labelled internal standards were monitored in negative-ion mode.

Supplementary Table S3. Sensitivity and method-validation results for phytohormone quantification in soybean leaves.

Limits of detection and quantification				
**Analyte**	**LOD (ng g^{-1} FW)**	**LOQ/LLOQ (ng g^{-1} FW)**	**LOD definition**	**LOQ/LLOQ definition**
tZ	0.5	1.5	S/N ≥ 3	S/N ≥ 10, accuracy 80–120%, and precision ≤20% RSD
IAA	1.0	3.0	S/N ≥ 3	S/N ≥ 10, accuracy 80–120%, and precision ≤20% RSD
ABA	2.0	5.0	S/N ≥ 3	S/N ≥ 10, accuracy 80–120%, and precision ≤20% RSD
GA_3	0.8	2.5	S/N ≥ 3	S/N ≥ 10, accuracy 80–120%, and precision ≤20% RSD

Extraction recovery and matrix effects							
**Analyte**	**QC level**	**Nominal concentration (ng g^{-1} FW)**	**Extraction recovery (%, mean ± SD)**	**Matrix factor**	**Matrix effect (%)**	**IS-normalized matrix factor**	**IS-normalized MF (%RSD)**
tZ	Low	4.5	84.6 ± 3.8	0.91	91	0.98	4.7
tZ	Medium	15	87.2 ± 3.1	0.94	94	1.00	3.9
tZ	High	60	89.1 ± 2.7	0.96	96	1.01	3.2
IAA	Low	9	82.8 ± 4.2	0.88	88	0.97	5.8
IAA	Medium	30	85.6 ± 3.6	0.91	91	0.99	4.6
IAA	High	120	87.9 ± 3.0	0.93	93	1.00	3.7
ABA	Low	15	86.4 ± 4.0	0.90	90	0.98	5.2
ABA	Medium	50	88.7 ± 3.3	0.93	93	1.00	4.1

ABA	High	200	90.2 ± 2.8	0.95	95	1.01	3.5
GA_3	Low	7.5	81.8 ± 4.5	0.87	87	0.96	6.2
GA_3	Medium	25	84.8 ± 3.7	0.90	90	0.98	4.9
GA_3	High	100	87.1 ± 3.1	0.92	92	0.99	4.0

Intra- and inter-day accuracy and precision

Analyte	QC level	Nominal concentration (ng g^{-1} FW)	Intra-day measured concentration (mean ± SD)	Intra-day accuracy (%)	Intra-day precision (%RSD)	Intra-day precision (%RSD)	Inter-day accuracy (%)	Inter-day accuracy (%)
tZ	LLOQ	1.5	1.47 ± 0.10	98	6.8	1.44 ± 0.12	96	8.3
tZ	Low	4.5	4.41 ± 0.27	98	4.5	4.37 ± 0.26	97	5.9
tZ	Medium	15	15.33 ± 0.54	102	3.5	15.15 ± 0.68	101	4.5
tZ	High	60	59.46 ± 2.05	99	3.4	58.80 ± 2.82	98	4.8
IAA	LLOQ	3	2.91 ± 0.23	97	7.9	2.88 ± 0.29	96	10.1
IAA	Low	9	9.18 ± 0.42	102	4.6	9.09 ± 0.55	101	6.1
IAA	Medium	30	29.42 ± 1.21	98	4.1	29.10 ± 1.63	97	5.6
IAA	High	120	121.20 ± 4.36	101	3.6	119.04 ± 5.83	99	4.9
ABA	LLOQ	5	5.15 ± 0.41	102	7.8	4.95 ± 0.49	99	9.9
ABA	Low	15	14.73 ± 0.72	98	4.9	14.55 ± 0.96	97	6.6
ABA	Medium	50	51.03 ± 2.04	102	4.0	50.50 ± 2.83	101	5.6
ABA	High	200	198.07 ± 6.73	99	3.4	196.00 ± 9.41	98	4.8
GA_3	LLOQ	2.5	2.43 ± 0.19	97	7.8	2.40 ± 0.24	96	10.0
GA_3	Low	7.5	7.65 ± 0.33	102	4.4	7.58 ± 0.46	101	6.1
GA_3	Medium	25	24.75 ± 0.97	99	3.8	24.50 ± 1.35	98	5.5
GA_3	High	100	101.01 ± 0.91	101	3.4	99.00 ± 4.65	99	4.7

Table note: FW, fresh weight; LOD, limit of detection; LOQ, limit of quantification; LLOQ, lower limit of quantification; QC, quality control; MF, matrix factor; IS, internal standard; RSD, relative standard deviation; S/N, signal-to-noise ratio. Extraction recovery and matrix effects were evaluated at low, medium, and high QC levels. Intra-day accuracy and precision were assessed using six independently prepared replicates at each QC level within one analytical batch, whereas inter-day accuracy and precision were evaluated across three independent analytical batches. Accuracy was calculated as the measured concentration divided by the nominal concentration × 100, and precision was expressed as the relative standard deviation. Acceptance limits were 85–115% for accuracy and ≤15% RSD for precision at QC levels above the LLOQ, and 80–120% and ≤20% RSD, respectively, at the LLOQ.
